# Neurobehavioral Responses of Rats Exposed to Low-Concentration Hexavalent Chromium [Cr(VI)] in Drinking Water Combined with a High-Fat Diet

**DOI:** 10.3390/app16157367

**Published:** 2026-07-22

**Authors:** Haiyan Lu, Samuel T. Vielee, Jessica Isibor, William J. Buchanan, Spencer H. Roof, Maitri Patel, Idoia Meaza, Aggie Brownell, Jennifer H. Toyoda, Sandra S. Diven, J. Calvin Kouokam, Rachel M. Wise, Rui Liu, Matthew Cave, Jamie Lynn Wise, John P. Wise

**Affiliations:** 1 Wise Laboratory of Environmental and Genetic Toxicology, Louisville, KY 40202, USA; 2 Department of Pharmacology and Toxicology, University of Louisville, Louisville, KY 40202, USA; 3 Department of Pharmaceutical Sciences, College of Pharmacy, University of New Mexico, Albuquerque, NM 87131, USA; 4 Division of Gastroenterology, Hepatology and Nutrition, University of Louisville, Louisville, KY 40202, USA; 5 Department of Pharmacology and Experimental Therapeutics, The University of Toledo, Toledo, OH 43614, USA

**Keywords:** hexavalent chromium, high-fat diet, neurobehavioral responses, drinking water exposures, Metallomics

## Abstract

Hexavalent chromium [Cr(VI)] is an established human carcinogen and was identified as the top chemical hazard in U.S. drinking water. Cr(VI) exposure has been associated with adverse neurological outcomes, while high-fat diet consumption is linked to cognitive impairment and neurodegenerative disease. However, limited evidence exists regarding their combined effects on brain health. This study investigated neurobehavioral changes and brain metal concentrations in young rats exposed to low concentrations of Cr(VI) in drinking water (0.05 mg/L or 0.1 mg/L) while fed a normal-fat or high-fat diet for 90 days. Cr(VI) exposure impaired grip strength in rats, while a high-fat diet attenuated this effect. Co-exposure to Cr(VI) with a high-fat diet reduced exploratory behavior and motor function, increased anxiety, and impaired spatial memory while also decreasing overall activity. Both sociability and social novelty preference were altered by the combined exposure in rats. Notably, Cr(VI) with high-fat diet co-exposure induced sex-specific behavioral changes. High-fat diet affected Cr brain accumulation, and Cr(VI) exposure and high-fat diet disrupted essential metal homeostasis in rat brains with sex differences. Jointly, the results suggest combined exposure to Cr(VI) and a high-fat diet may increase the risk of adverse neurological outcomes.

## 1. Introduction

Chromium (Cr) is a naturally occurring element abundant in the Earth’s crust, primarily existing in two oxidation states: trivalent chromium [Cr(III)] and hexavalent chromium [Cr(VI)] [1]. These oxidation states differ significantly in their chemical properties and toxicokinetic features: Cr(III) may impart pharmacological benefits by influencing glucose and lipid metabolism [2], whereas Cr(VI) is highly toxic to most organs, with known links to lung cancer, liver and kidney dysfunction, reproductive toxicity, developmental abnormalities, and respiratory diseases [3]. Despite its toxicity, Cr(VI) is extensively used in various industrial activities such as the production of chrome pigments, chrome plating, stainless steel manufacturing, and leather tanning due to its hardness, bright color, electrical conductivity, and good corrosion resistance [4]. Cr(VI) is classified by the International Agency for Research on Cancer as a known human carcinogen (Group 1A) that affects the lungs, liver, and kidneys [3,5,6]. Reflecting its significant health risks, the U.S. Agency for Toxic Substances and Disease Registry ranks Cr(VI) as 17th on its Substance Priority List as one of the greatest chemical threats to human health [7].

However, there is a paucity of evidence regarding the health effects following Cr(VI) ingestion. Chromate contamination of drinking water has been a longstanding concern, primarily resulting from industrial wastewater discharges and the use of chromates as rust inhibitors. Individuals relying on well water are particularly vulnerable due to aquifer contamination. A study conducted in China reported increased gastric cancer mortality in areas with elevated Cr(VI) levels (>0.5 mg/L) in drinking water [8]. A study in the Oinofita region of Greece showed a significant increase in the incidence of liver, lung, kidney, and other urogenital cancers in areas where water was contaminated with Cr(VI) (up to 0.156 mg/L) [9]. In 2008, the U.S. National Toxicology Program released a two-year rodent study showing that Cr(VI) is carcinogenic when ingested, with male and female mice developing significantly increased small intestinal tumors in drinking water concentrations of 85.7 mg/L and 57.3 mg/L, respectively [10]. More recently, a systematic review of epidemiological and risk-assessment studies concluded that exposure to Cr(VI)-contaminated drinking water is associated with increased carcinogenic risk in humans [11].

The main sources of oral Cr(VI) exposure are food and drinking water. Dietary exposure to Cr(VI) through food remains uncertain due to limited data on the specific chemical forms present. Reported total Cr levels, including both Cr(VI) and Cr(III), in most foods range from less than 0.01 to 1.3 mg/kg [12]. In uncontaminated water, Cr concentrations are generally low, ranging from 0.001 to 0.01 mg/L in rivers and lakes [3]. Drinking water contamination by Cr(VI) originates from two primary sources: contaminated groundwater and aging cast iron pipes. Cr(VI) in groundwater can originate from natural sources or industrial pollution. Natural sources most often arise from the oxidation of Cr(III) to Cr(VI) by manganese oxides in mafic and ultramafic rocks and serpentine soils [13–15]. Industrial activities, however, have significantly increased Cr(VI) releases into soil, groundwater, and air. In 2009, domestic manufacturing and processing facilities discharged an estimated 220,474 kg of Cr(VI) compounds into surface waters [3]. Within public drinking water distribution systems, cast iron pipes are used in 57% of systems and exhibit iron corrosion scales with extended use [16]. Initially, Cr(III) adsorbs to the iron corrosion scales, becoming a sink for drinking water Cr. However, with a shift in water chemistry to more acidic conditions (e.g., pH 6.0), this stored Cr is oxidized and released back into the water supply as Cr(VI) [17]. This oxidation process is further catalyzed by phosphates, chlorine, and bromine, which are common water disinfectants. Altogether, these studies describe a public health crisis for Cr(VI) in U.S. drinking water.

Public concern over Cr(VI) contamination in drinking water was amplified by the 1994 case led by Erin Brockovich (and again in 2000 after a blockbuster film with her name), which highlighted Cr(VI) contamination in Hinkley, California, and resulted in a $333 million settlement. Since then, more than 30 cities across the U.S. have reported elevated Cr(VI) levels in their drinking water, often exceeding regulatory limits [18]. Currently, the U.S. Environmental Protection Agency (EPA) and the World Health Organization (WHO) set drinking water standards for total Cr at 0.1 mg/L and 0.05 mg/L, respectively, but neither has established specific standards for Cr(VI) [12,19]. However, studies report a near 1:1 ratio for total Cr to Cr(VI) in drinking water [15,20]. A recent survey of national drinking water data from systems serving over 90% of the U.S. population revealed that Cr(VI) poses the highest exposure risk, with levels exceeding its health reference threshold in 94% of the population studied [20].

Although human studies on Cr(VI) neurotoxicity are limited, epidemiological and case studies have linked Cr(VI) exposure to neurological effects such as memory impairment, autism spectrum disorder, and motor neuron disease [5,21]. Rodent models have expanded on these effects, reporting that Cr accumulates in the brain, particularly in the hypothalamus and hippocampus, and disrupts essential metal homeostasis [22–24]. Recent studies have further demonstrated that Cr(VI) causes behavioral changes, oxidative stress, and neuroinflammation in rodents [25–29].

High-fat diet impacts brain health, especially cognitive decline, and increases the risk of neurodegenerative diseases such as Alzheimer’s disease [30]. Epidemiological studies indicate that long-term high-fat diet consumption contributes to cognitive impairment [31]. High saturated fat intake is associated with deficits in memory, attention, and mental flexibility. A study in middle-aged adults reported a higher risk of cognitive impairments, including memory and processing speed, linked to saturated fat consumption [32]. Another study with a 6-year follow-up reported long-term intake was correlated with declines in memory and attention [33]. Notably, these cognitive effects are more pronounced in older adults [34]. Even short-term high-fat diet consumption in humans can impair cognitive functions, such as attention and mood [35,36]. Similarly, short-term (as short as three days) consumption of high-fat diet in rats can lead to memory deficits [37]. High saturated fat intake is associated with an increased risk of Alzheimer’s disease [30]. Animal studies demonstrate high-fat diet exacerbates brain Aβ pathology, a key hallmark of Alzheimer’s disease, and leads to cognitive decline, tau hyperphosphorylation, and microglial activation [30,38,39]. High-fat diet impairs cognition by inducing low-grade inflammation, oxidative stress, and insulin resistance [40,41]. These changes disrupt synaptic plasticity, which is critical for learning and memory [42].

Human exposure to metals has risen significantly in recent decades, driven by industrialization and socioeconomic shifts that have also increased the prevalence of high-fat diet consumption. Both metal exposure and high-fat diets are recognized health risk factors, particularly in relation to cognitive impairment [43]. Studying the effects of high-fat diet and metal exposure on brain health has become an urgent matter. High-fat diet combined with exposure to metals like aluminum, lead, cadmium, and arsenic was reported to significantly impair cognitive function, disrupt memory, and accelerate neurodegeneration [43–45]. A recent study revealed that cadmium exposure combined with high-fat diet disrupts essential metal homeostasis in the mouse brain, with the high-fat diet further elevating cadmium levels. These exposures also led to increased superoxide levels in the cortex, amygdala, and hippocampus [46].

To date, little is known about the combined effects of Cr(VI) exposure and a high-fat diet on brain health, which is a major data gap requiring urgent attention. We hypothesized that high-fat diet would modify the neurobehavioral and neurochemical effects of Cr(VI), resulting in altered behavioral outcomes, brain Cr accumulation, and essential metal homeostasis in a sex-dependent manner. Therefore, in this study, we examined neurobehavioral changes, determined brain Cr accumulation, assessed essential metal homeostasis, and investigated sex-specific responses in rats exposed to low-concentration Cr(VI) (0.05 mg/L or 0.1 mg/L) in drinking water while being fed a normal-fat or high-fat diet.

## 2. Materials and Methods

### 2.1. Animal Models of Sodium Chromate Exposure

Male and female Sprague-Dawley rats (Envigo, Indianapolis, IN, USA) were enrolled in the study at 12 weeks of age and maintained under standard laboratory conditions. All animals were housed two per cage under standard ambient room temperature, relative humidity, a 12:12 h light–dark cycle, and provided food and water ad libitum. Animal procedures were approved by the Institutional Animal Care and Use Committee of the University of Louisville (#21934), and followed the appropriate guidelines set forth by the American Veterinary Medical Association. The study design is shown in Figure 1. A total of 96 Sprague-Dawley rats were used in the study, including 48 males and 48 females. Rats were randomly assigned to one of six treatment groups (*n* = 8/sex/group) defined by diet (normal-fat diet or high-fat diet) and drinking water Cr(VI) concentration (0, 0.05, or 0.1 mg/L). Endpoints where the sample size decreased due to unavailable data points or outlier removal are reported in the associated figure legend.

Rats were randomly divided into two diet groups: a normal-fat diet (normal diet) group and a high-fat diet group; and 3 drinking water groups: tap water, 0.05 mg Cr(VI)/L in tap water, or 0.1 mg Cr(VI)/L in tap water, reflecting maximum contaminant levels set by the WHO and U.S. EPA, respectively. Rats assigned to the normal-fat diet group were fed a purified ingredient diet containing 10 kcal% fat, 20 kcal% protein, and 70 kcal% carbohydrate (D12450Ji, Research Diets, New Brunswick, NJ, USA; energy density 3.82 kcal/g). Rats assigned to the high-fat diet group were fed a purified ingredient diet containing 60 kcal% fat, 20 kcal% protein, and 20 kcal% carbohydrate (D12492, Research Diets, New Brunswick, NJ, USA; energy density 5.24 kcal/g). Cr(VI) was administered as sodium chromate (307831, Sigma Aldrich, St. Louis, MO, USA) dissolved in tap water, and the concentration was adjusted to reflect Cr(VI) content. Weekly Cr(VI) exposures were prepared from 1000× stock solutions in tap water, with 50 mg Cr(VI)/L and 100 mg Cr(VI)/L stocks diluted to final concentrations of 0.05 mg Cr(VI)/L and 0.1 mg Cr(VI)/L, respectively. Body mass, water consumption, and food consumption were monitored weekly. For quality assurance and control, drinking water samples were collected weekly for metal analysis to confirm Cr(VI) concentrations.

### 2.2. Neurobehavior Assays

Rats participated in one behavior assay per week, including the grip strength, open-field assay, elevated plus maze assay, Y-maze, and 3-chamber assay (Table 1), as reported previously [28,29]. These tests were spaced to ensure adequate recovery time and minimize stress, with at least 7 days between assays for each rat. Then 70% ethanol was used to sanitize and deodorize the equipment between consecutive rats. Various chambers used in the corresponding assays were built by Dr. Jun Cai.

All behavior assays were conducted in the same room under red light conditions, with foam boards enclosing the area to minimize ambient noise and block the rat’s view of the experimenter. The assays were performed in the afternoon, coinciding with the light phase of the 12 h light–dark cycle, to reduce variability due to circadian rhythms. Each behavior was assessed twice throughout the study, with testing intervals spaced evenly over time.

For video recording, aerial and side views of the assays were captured using a 4 mm C Series Fixed Focal Length Lens camera (33300, Edmund Optics, Barrington, NJ, USA) and a 6 mm C Series Fixed Focal Length Lens camera (33301, Edmund Optics), respectively. Custom programs created in LabVIEW 2019 (v.190fl, National Instruments, Austin, TX, USA) were used to record and analyze data for open-field assay, elevated plus maze, Y-maze, and 3-chamber assay.

### 2.3. Grip Strength

Grip strength was measured using an ALMEMO Universal Measuring Instrument (Holzkirchen, Germany, 24910) during weeks 1 and 7 of the exposure period. After gently lowering the rat’s forefeet to grip a horizontal bar (1.016 mm in diameter and 6.35 cm in width), each rat was held horizontally by the base of its tail and pulled backward at a steady rate until it released its grip on the bar. Grip strength was measured in triplicate for each rat, with the peak force from each trial recorded. Only measurements where the rat released its grip from both forefeet simultaneously were considered valid. Rats that refused to grip the bar after 10 attempts were marked as noncompliant, and no measurement was recorded. Median values from the three trials were used for analysis and comparisons between study groups.

### 2.4. Open-Field Assay

Open-field assay was conducted during weeks 2 and 8 of Cr(VI) exposure. The chamber, measuring 71 cm × 71 cm × 43 cm (length × width × height), consisted of a square open area enclosed by four walls, one of which was transparent to allow side-view camera recording. Rats were individually placed at the center of the chamber and allowed to explore freely for 10 min while their behavior was recorded from both aerial and side views. Behavior recordings were analyzed for changes in center area exploration (an indicator of anxiety), distance traveled (locomotor function), and rearing behavior (motor coordination) [47]. Rearing counts were manually scored from blinded video recordings (i.e., video files were renamed and sequence mixed), with a rearing event defined as the rat standing on its hind legs and lifting its forefeet above its center of mass. A rearing event was considered complete when the rat returned both forefeet to the chamber floor.

### 2.5. Elevated Plus Maze

The elevated plus maze assay was conducted during weeks 3 and 9 of Cr(VI) exposure. The maze consisted of two open arms (without walls) and two closed arms (surrounded by 43 cm high walls on three sides) arranged in a “+” shape and placed on a stand 74.5 cm above ground. The dimensions of each arm were 51 cm × 15 cm (length × width), and the center of the maze was a 15 cm × 15 cm (length × width) square. Rats were individually placed in the center of the maze, facing an open arm, and allowed to explore freely for 5 min while their behavior was recorded from an aerial perspective. Anxiety levels were assessed based on the percentage of time spent in the open arms [48], with decreased exploration of the open arms indicating increased anxiety.

### 2.6. Y-Maze

The Y-maze assay was conducted during weeks 4 and 10 of Cr(VI) exposure. The maze consisted of three radial arms arranged in a “Y” shape (length × width × height: 51 cm × 15 cm × 43 cm), each fully enclosed by walls and connected by an equilateral triangle center (side length: 15 cm). Rats were individually placed at the center of the maze and allowed to freely explore for 8 min, with behavior recorded from an aerial view. Spatial memory was assessed based on non-alternating arm entries, while general activity was evaluated by the frequency of arm entries [49]. Behavior was analyzed by tracking the sequence of arm entries to determine the number of alternations and non-alternations. An alternation was defined as sequential entries into all three arms, while a non-alternation was recorded when any arm was re-entered within a sequence of three entries. Arm entries were counted when all four paws crossed from the center triangle into an arm. An increase in non-alternations reflects impaired spatial memory. The percentage of non-alternations was calculated using the formula:

(1) $$\text{Percentage of Non-Alterations}=\frac{\left(\text{Number of Non-Alternations}\right)}{\left(\text{Number of Alternations}+\text{Number of Non-Alternations}\right)}\times 100$$

General activity was determined by measuring arm entries per minute. An entry was counted when all limbs left the center triangle and entered an arm. Increased arm entries per minute indicated hyperactivity, while decreased frequency suggested hypoactivity.

### 2.7. 3-Chamber Assay

The 3-chamber assay was conducted during weeks 5 and 11 of Cr(VI) exposure. The apparatus consisted of an enclosed space (length × width × height: 122 cm × 82 cm × 43 cm), with two interior walls and dividers creating three chambers (length × width × height: 41 cm × 82 cm× 43 cm). The exterior chambers housed weighted inverted baskets to secure reference rats (a cagemate or a stranger), while the center chamber was left empty. The baskets used prevented direct contact between the rats. This test evaluates the free choice of test animals to spend time in one of three chambers, interacting with a cagemate, an empty cage, or a stranger placed under a basket [50]. Test rats were initially placed in the center chamber, and the dividers were removed to allow free exploration of the entire space and interaction with the reference rats under the baskets. Each test consisted of three trials (10 min per trial), with behavior recorded from aerial and side cameras. Trial 1 served as an acclimation phase with no additional rats present. In Trial 2, a cagemate was placed under one basket, and in Trial 3, a stranger rat was placed under the other basket. Recordings were manually analyzed to count chamber entries and interactions, with researchers blinded by renaming video files and the sequence mixed. Direct interactions were defined as the test rat touching or climbing on the baskets, while indirect interactions were defined as the test rat extending its body within 5 cm of a basket. The ratio of total interactions between the cagemate and the empty cage, as well as between the cagemate and the stranger rat, was assessed to determine the effects of Cr(VI) exposure.

### 2.8. Sample Collection and Microdissection of Brain Regions

After the 90-day study was completed, rats were anesthetized via intraperitoneal injection of a 100 mg/mL ketamine and 100 mg/mL xylazine (combined at a 9:1 ratio) at a dose of 0.1 mL/kg bodyweight. Blood was collected, then rats were euthanized by exsanguination using cardiac perfusion with 250–300 mL of cold 1× Dulbecco’s Phosphate-Buffered Saline (DPBS) (without calcium or magnesium) (Corning, New York, NY, USA, 20–031-CV) vacuum-filtered through a 0.22 μm membrane. After perfusion, the rats were decapitated, and their brains were carefully extracted. The extracted brains were rinsed in ice-cold 1× DPBS and gently blotted to remove excess liquid. Brains were then bisected using a Rodent Brain Matrix (RBM-4000C, ASI Instruments, Warren, MI, USA) on ice. One hemisphere was microdissected to isolate specific regions: the brainstem, cortex, cerebellum, hippocampus, hypothalamus, and striatum. Whole-brain mass and individual brain region masses were measured and recorded. The cortex was further divided into the rostral and caudal cortex, with metal levels from the rostral cortex reported in this study. Samples for metal analysis were collected into trace element-free tubes.

### 2.9. Metal Analysis by Inductively Coupled Plasma–Mass Spectrometry

Inductively coupled plasma–mass spectrometry (ICP-MS) was performed following a previously published method [24]. Tissues were digested in 70% nitric acid at 85 °C for 3 h. After the initial acid digestion, samples were cooled to room temperature and subjected to further digestion with 3% hydrogen peroxide for 1 h at room temperature. After digestion, samples were diluted with Millipore water to achieve a final concentration of 5% nitric acid and filtered through Acrodisc 32 mm, 0.45-μm Supor^®^ filters (4654, Pall Corporation, Port Washington, NY, USA). Samples were analyzed using an Agilent 7900 ICP-MS, with analyses performed at the Metallomics Core Facility of the University of Louisville. Certified reference standards for trace elements in water (NIST 1643f, Millipore Sigma, Burlington, MA, USA) were included to ensure accuracy. Quality control measures included the use of initial calibration blanks and initial calibration verification standards, which were analyzed at the beginning of the run, after every 10 samples, and at the end of the run. An internal standard was incorporated into all sample runs to correct for potential matrix effects. Any sample concentrations below the detection limit were reported as one-half the limit of detection.

### 2.10. Statistical Analyses

In all datasets, we first considered our data with sexes pooled, then considered each sex independently. We did not make statistical comparisons between sexes, but commented on differences in effects (e.g., behavior relative to controls was increased in males and decreased in females). Statistical analyses were performed using GraphPad Prism version 10.4.0. All data were expressed as mean ± standard error of the mean (SEM). In behavior data analyses, outliers were removed after being identified by the Iterative Grubb’s Test and ROUT test (α = 0.05), where both tests agreed. For metals data, outliers were excluded from analyses if a data point was ten times higher than the mean for the rest of the group. To evaluate the independent and combined effects of diet and Cr(VI) exposure, behavioral outcomes were analyzed using two-way ANOVA with Diet and Cr(VI) concentration as fixed factors. Pairwise comparisons were used to identify specific group differences and aid biological interpretation, whereas conclusions regarding overall Cr(VI) effects were based primarily on the two-way ANOVA results. Based on the normality test results, either an unpaired *t*-test with or without Welch’s correction or the Mann–Whitney test was used to assess statistical differences in Cr(VI) concentrations or diets. Statistical significance was determined as a *p* value less than 0.05, but we also noted *p* values less than 0.1 as “notable” to indicate where follow-up studies with stronger effect sizes may find significant differences.

## 3. Results

### 3.1. Cr(VI) Impaired Grip Strength in Rats, While a High-Fat Diet Co-Exposure Mitigated This Effect

We evaluated the effects of Cr(VI) exposure in drinking water and high-fat diet feeding on grip strength in rats after 1 and 7 weeks. Two-way ANOVA revealed no significant main effects of diet or Cr(VI) exposure and no significant Diet × Cr(VI) interaction for grip strength after 1 week of exposure (Supplementary Table S1). After 7 weeks, a significant main effect of diet was observed (F(1, 84) = 14.15, *p* = 0.0003), whereas neither Cr(VI) exposure nor the Diet × Cr(VI) interaction reached statistical significance (Supplementary Table S1). We first considered our data with males and females combined. After 1 and 7 weeks of exposure, we observed that the grip strength of rats exposed to Cr(VI) in the normal-fat diet groups decreased. High-fat diet alone did not impact grip strength relative to normal-fat diet alone. In contrast, rats exposed to Cr(VI) while receiving a high-fat diet exhibited grip strengths comparable to high-fat diet controls and generally higher than those of the corresponding Cr(VI)-exposed normal-fat diet groups, suggesting that high-fat diet feeding attenuated the reduction in grip strength associated with Cr(VI) exposure (Figure 2A,D). We then considered each sex independently. We observed after 1 week of Cr(VI) exposure the grip strength of male rats in the normal-fat diet group decreased, while the grip strength of female rats exhibited no differences (Figure 2B,C). After 1 week of Cr(VI) exposure with high-fat diet, the grip strength of female rats exposed to Cr(VI) increased slightly relative to those receiving tap water; whereas there was no apparent Cr(VI) effect in males fed high-fat diet (Figure 2B,C). After 7 weeks of exposure, the grip strength of male rats in the normal-fat diet group exposed to 0.1 mg/L Cr(VI) exhibited a notable decrease, while the grip strength of female rats lacked an effect from Cr(VI) exposure (Figure 2E,F). Specifically, mean grip strengths were 407.0, 379.6, and 322.1 g for male control, 0.05, and 0.1 mg/L Cr(VI), and 287.3, 280.3, and 268.9 g for female control, 0.05, and 0.1 mg/L Cr(VI), respectively. Interestingly, after 7 weeks of exposure, male rats receiving Cr(VI) together with a high-fat diet exhibited significantly greater grip strength than the corresponding Cr(VI)-exposed normal-fat diet groups, suggesting that the high-fat diet may attenuate the reduction in grip strength associated with Cr(VI) exposure. Similarly, female rats exposed to 0.05 mg/L Cr(VI) while receiving a high-fat diet exhibited significantly greater grip strength than the corresponding normal-fat diet group (Figure 2E,F). Whereas females exposed to 0.1 mg/L Cr(VI) with high-fat diet exhibited impaired grip strength compared with the high-fat diet group drinking tap water and no difference relative to the 0.1 mg/L Cr(VI) with normal-fat diet (Figure 2F). Specifically, mean grip strengths were 437.1, 473.4, and 463.1 g for male control, 0.05, and 0.1 mg/L Cr(VI) combined with high-fat diet, and 347.9, 355.8, and 293.1 g for female control, 0.05, and 0.1 mg/L Cr(VI) combined with high-fat diet, respectively. Furthermore, we observed that after 7 weeks of exposure, the grip strength of female rats fed a high-fat diet with tap water or 0.05 mg/L Cr(VI) was significantly higher than that of the normal-fat diet group (Figure 2F).

### 3.2. Cr(VI) with High-Fat Diet Co-Exposure Decreased Exploratory Behavior and Locomotor Activity in Rats

Rats underwent the open field assay during weeks 2 and 8 of the study. Exploratory behavior was evaluated based on the percentage of time spent in the center area of the open field chamber (Figure 3), motor coordination was assessed through rearing behavior (Figure 4), and motor function was measured by the distance traveled (Figure 5). Two-way ANOVA revealed no significant main effects of diet, Cr(VI), or Diet × Cr(VI) interaction for center area exploration at either week 2 or week 8 (Supplementary Table S1). Similarly, no significant main effects or interaction effects were identified for rearing behavior at either time point (Supplementary Table S1). In contrast, a significant Diet × Cr(VI) interaction was observed for distance traveled at week 2 (F(2, 86) = 4.340, *p* = 0.0160), indicating that the effect of Cr(VI) on locomotor activity depended on dietary condition. A significant main effect of diet was also observed for distance traveled at week 8 (F(1, 90) = 7.930, *p* = 0.0060) (Supplementary Table S1).

We evaluated the center area exploration of rats after 2 and 8 weeks of exposure. We first considered our data with males and females combined. After 2 weeks of drinking water Cr(VI) exposure, there was no significant change in the center area exploration of rats in the normal-fat diet group, and the high-fat diet slightly reduced the center area exploration of rats in the 0.1 mg/L group (Figure 3A). Then we considered the center area exploration within each sex (Figure 3B,C). Cr(VI) alone slightly increased center exploration in males exposed to 0.05 mg/L and in females exposed to 0.1 mg/L, whereas high-fat diet with Cr(VI) co-exposure reduced the center area exploration of female rats but not male rats (Figure 3B,C). Specifically, the average percent time of center area exploration was 13.0, 15.5, and 17.8% for female control, 0.05, and 0.1 mg/L Cr(VI) combined with normal-fat diet, and 18.1, 13.5, and 11.5% for female control, 0.05, and 0.1 mg/L Cr(VI) combined with high-fat diet, respectively. After 8 weeks of drinking water Cr(VI) exposure, we observed no significant change in the center area exploration of rats (sexes pooled) in the normal-fat diet group, while Cr(VI) notably reduced the central area exploration of rats in the high-fat diet group in a concentration-associated manner (Figure 3D). Similar results were observed when analyzing different sex groups, though lacking statistical significance (Figure 3E,F).

We evaluated the rearing behavior of rats after 2 and 8 weeks of exposure. When we considered effects with sexes pooled, there were no significant changes in the rearing behavior of rats for either diet group or Cr(VI)-exposed groups after 2 or 8 weeks of exposure to Cr(VI) in drinking water (Figure 4A,D). At the 2-week time point, there were no significant changes in the rearing behavior of rats in different sex groups; however, we noted a general reduction in the rearing behavior in females fed a high-fat diet relative to normal-fat diet groups, except when exposed to 0.05 mg/L Cr(VI) in drinking water (Figure 4B,C). After 8 weeks of exposure, high-fat diet with tap water increased the rearing behavior of male rats, but not high-fat diet with Cr(VI) exposure (Figure 4E). There was no change in the rearing behavior of female rats after 8 weeks of exposure (Figure 4F).

We evaluated the effects of Cr(VI) and high-fat diet co-exposure for 2 and 8 weeks on the distance traveled in rats. After 2 weeks of exposure, 0.05 mg/L Cr(VI) in the normal-fat diet group significantly reduced the distance traveled in pooled sexes (Figure 5A), with this effect being particularly evident in males (Figure 5B). However, the high-fat diet reversed this effect in the distance traveled in female rats exposed to 0.05 mg/L Cr(VI) and these females exhibited an increase on distance traveled (Figure 5C). After 8 weeks of exposure, there was an overall reduction in distance traveled in all high-fat diet groups (Figure 5D). In the normal-fat diet group, 0.1 mg/L Cr(VI) slightly increased the distance traveled by rats, but the co-exposure to high-fat diet and 0.1 mg/L Cr(VI) significantly reduced the distance traveled in both male and female rats, relative to normal-fat diet (Figure 5D–F).

### 3.3. High-Fat Diet Altered the Effects of Cr(VI) on Anxiety-like Behavior in Rats

We evaluated the anxiety-like behavior of rats in the elevated plus maze after 3 and 9 weeks of co-exposure to Cr(VI) with a high-fat diet. Two-way ANOVA revealed a significant main effect of diet on open-arm exploration after 3 weeks of exposure (F(1, 90) = 4.063, *p* = 0.0468), whereas neither Cr(VI) exposure nor the Diet × Cr(VI) interaction reached statistical significance (Supplementary Table S1). No significant main effects or interaction effects were observed after 9 weeks of exposure (Supplementary Table S1). After 3 weeks of exposure, 0.1 mg/L Cr(VI) in the normal-fat diet group slightly increased open arm exploration, particularly in males (Figure 6A–C), suggesting reduced anxiety-like behavior under normal-fat diet conditions. In contrast, although high-fat diet alone increased open arm exploration relative to normal-fat diet controls, Cr(VI) co-exposure produced a concentration-dependent decrease in open arm exploration relative to the high-fat diet control group (Figure 6A). These findings indicate that the effects of Cr(VI) on anxiety-like behavior were dependent on dietary condition, with Cr(VI) tending to decrease anxiety-like behavior under normal-fat diet conditions but increase anxiety-like behavior in rats receiving a high-fat diet. Notably, the increase observed in high-fat diet control was significant in male rats but negligible in females (Figure 6B,C). Specifically, the average percent times of open arm exploration were 27.8, 28.2, and 35.3% for sexes pooled control, 0.05, and 0.1 mg/L Cr(VI) combined with normal-fat diet, and 38.4, 34.9, and 32.6% for sexes pooled control, 0.05, and 0.1 mg/L Cr(VI) combined with high-fat diet, respectively. After 9 weeks of exposure, 0.05 mg/L Cr(VI) in the normal-fat diet group slightly increased open arm exploration behavior of rats, which was more prominent in males than females (Figure 6D–F). After both exposure weeks, 0.1 mg/L Cr(VI) decreased open arm exploration behavior of rats in the high-fat diet group, and it was significantly decreased in the female rat group after 9 weeks of exposure (Figure 6F).

### 3.4. Cr(VI) and High-Fat Diet Co-Exposure Produced Modest Changes in Spatial Memory and Activity-Related Behaviors in Rats

Rats were assessed with a Y-maze test after 4 and 10 weeks of co-exposure to Cr(VI) and a high-fat diet. Non-alternating exploration was used as a measure of spatial memory (Figure 7) and arm entry frequency was used as a measure of activity (Figure 8). Two-way ANOVA did not identify significant main effects of diet, Cr(VI), or Diet × Cr(VI) interactions for non-alternating exploration at either week 4 or week 10 (Supplementary Table S1). Similarly, no significant main effects or interaction effects were observed for arm entry frequency at either time point (Supplementary Table S1).

When sexes were pooled, we observed no change in non-alternating exploration in rats after 4 weeks of exposure (Figure 7A). When sex was considered, we observed a slight concentration-associated increase in non-alternations by male rats with normal diet; high-fat diet controls exhibited a slight increase relative to normal-fat diet controls, but we observed no effects in the high-fat diet co-exposure groups (Figure 7B). Female rats in the high-fat diet group exhibited fewer non-alternations in the control and 0.05 mg/L Cr(VI) groups (Figure 7C), whereas within the high-fat diet female groups, pairwise comparisons indicated increased non-alternations in rats exposed to 0.1 mg/L Cr(VI) relative to controls (Figure 7C). After 10 weeks, there were no significant differences in non-alternations between the normal-fat diet and the high-fat diet groups (Figure 7D–F). However, in the high-fat diet group (sexes pooled), Cr(VI) slightly increased non-alternations in the 0.05 mg/L Cr(VI) group but slightly decreased non-alternations in the 0.1 mg/L Cr(VI) group (Figure 7D); this increase was more prominent in females (Figure 7F), while this decrease was more prominent in males (Figure 7E).

We evaluated the number of arm entries per minute in the Y-maze after 4 and 10 weeks to assess each rat’s relative activity. With sexes pooled, we observed a slight increase in the number of arm entries per minute in the normal-fat diet with 0.1 mg/L Cr(VI) group after 4 weeks, while Cr(VI) decreased the number of arm entries per minute within the high-fat diet group (Figure 8A). Notably, the high-fat diet control group was significantly higher than the normal diet control group (Figure 8A). Specifically, the average numbers of arm entries per minute were 3.7, 3.7, and 4.0 for sexes pooled control, 0.05, and 0.1 mg/L Cr(VI) combined with normal-fat diet, and 4.1, 3.7, and 3.6 for sexes pooled control, 0.05, and 0.1 mg/L Cr(VI) combined with high-fat diet, respectively. When we considered sexes separately, we observed no significant effects in females, regardless of diet or Cr(VI) exposure (Figure 8C). Whereas male rats exhibited more arm entries (hyperactivity) in high-fat diet controls than normal diet controls, and within the high-fat diet groups we observed significantly reduced arm entries (hypoactivity) with Cr(VI) exposure (Figure 8B). After 10 weeks, regardless of sex, we observed a slight concentration-associated increase in arm entries in the normal diet groups but a slight concentration-associated decrease in the high-fat diet groups (Figure 8D). Notably, the high-fat diet control group was slightly higher than the normal diet control group. Considering sexes separately, we observed a similar pattern with the data in both sexes, though females overall exhibited more arm entries than males (Figure 8E,F).

### 3.5. Cr(VI) and High-Fat Diet Co-Exposure Affected Sociability and Social Novelty Preference in Rats

Sociability and social novelty preference were assessed in rats using the 3-chamber assay after 5 and 11 weeks of exposure. To assess sociability, we considered (1) the number of interactions between the test rat and its cagemate, (2) the number of interactions between the test rat and an empty cage, and (3) the ratio of cagemate to empty cage interactions (Figure 9, Supplementary Figure S1). Social novelty preference was assessed by (1) the number of interactions between the test rat and its cagemate, (2) the number of interactions between the test rat and a stranger, and (3) the ratio of cagemate to stranger interactions (Figure 10, Supplementary Figure S2). Here, it is not only important to consider how Cr(VI), diet, or sex impacts the number of interactions with a stranger/empty cage or cagemate, but also to consider how the numbers of interactions with a stranger/empty cage or cagemate compare to each other within a study group. A similar number of interactions between a cagemate and an empty cage may indicate a lack of sociability, whereas a similar number of interactions between a cagemate and a stranger may indicate a lack of recognition/distinction between the two. Two-way ANOVA of sociability and social novelty ratios did not identify significant main effects of diet, Cr(VI), or Diet × Cr(VI) interactions (Supplementary Table S1). However, several trends were observed. For sociability, a trend toward a Diet × Cr(VI) interaction was detected at week 5 (F(2, 84) = 2.860, *p* = 0.0629). In addition, Cr(VI) exposure exhibited trend-level effects on sociability at week 11 (*p* = 0.0907) and on social novelty preference at week 11 (*p* = 0.0573).

We first considered the effects of Cr(VI) and diet on the sociability of rats after 5 and 11 weeks of exposure. We observed rats generally preferred to interact with cagemates rather than with an empty cage, and no statistically significant differences were observed in the interactions with cagemates or empty cages, regardless of Cr(VI) or diet (Figure 9A–C). However, after 5 weeks, a concentration-associated pattern emerged in the normal diet groups exposed to Cr(VI), where cagemate interactions decreased while empty cage interactions increased, resulting in the two measures becoming more similar (Figure 9A). This pattern was more apparent in males than females. At 0.1 mg/L Cr(VI) groups, the difference between cagemate and empty cage interactions was smaller in normal diet animals than in high-fat diet animals, suggesting a diet-dependent effect, with HFD restoring sociability in males (Figure 9B). In females, cagemate interactions remained relatively stable in the normal diet groups but decreased in the high-fat diet groups with increasing Cr(VI), suggesting HFD made females less social and indicating a distinct sex- and diet-specific response (Figure 9C). Further, we observed a slight concentration-associated decrease in cagemate interactions for high-fat diet-fed females that was absent in normal diet-fed females or in high-fat diet-fed males (Figure 9C). When we consider these data as ratios of cagemate interactions to empty cage interactions for each rat (cagemate/empty cage), we observed the normal diet group exhibited a decreased ratio after exposure to Cr(VI); the mean ratios for the control, 0.05, and 0.1 mg/L Cr(VI) groups were 2.5, 1.7, and 1.4, respectively (Supplementary Figure S1A). The high-fat diet controls exhibited a lower ratio than the normal diet controls, and Cr(VI) exposure in the high-fat diet groups induced an increased ratio compared to the normal-fat diet group. The mean ratios for the high-fat diet control, 0.05, and 0.1 mg/L Cr(VI) groups were 2.0, 1.9, and 2.3, respectively (Supplementary Figure S1A). This change in ratio was pronounced in male rats, but not in female rats (Supplementary Figure S1B,C). There was a less apparent pattern after 11 weeks for all groups, nearly identical in the normal diet group exposed to 0.1 mg/L Cr(VI), while high-fat diet females showed greater separation between the two measures. Curiously, comparable interactions were observed in high-fat diet control females between the cagemate and empty cage, with increasing Cr(VI) exposure creating more disparity between these types of interactions (Figure 9F). When we consider these data as ratios for each rat, we observed the ratio increased in males after exposure to 0.05 mg/L Cr(VI) in both diet groups (Supplementary Figure S1E). We observed that these ratios in female rats decreased in the normal diet group while the high-fat diet group showed the opposite effect, and the high-fat diet controls had lower values than the normal diet controls (Supplementary Figure S1E,F).

We then assessed the rats’ social novelty preference by comparing interactions with a cagemate vs. a stranger rat. Overall, rats preferred to interact with a stranger rat, rather than a cagemate. Importantly, the difference between cagemate and stranger interactions varied by diet and Cr(VI) exposure. We observed a notable difference between the normal diet and high-fat diet groups when sexes were pooled: the high-fat diet control group exhibited more interactions with strangers than the normal diet control group, though interactions with a cagemate were similar (Figure 10A). With increasing Cr(VI) exposure, stranger interactions changed more prominently than cagemate interactions in the normal diet groups. Specifically, in normal diet groups, stranger interactions increased with Cr(VI), whereas in high-fat diet groups, stranger interactions decreased at 0.1 mg/L, reducing the separation between cagemate and stranger interactions (Figure 10A). This pattern was more apparent in females than in males (Figure 10B,C). In males fed a high-fat diet, exposure to 0.1 mg/L Cr(VI) increased cagemate interactions (green bars) relative to controls, resulting in similar levels of cagemate and stranger interactions, suggesting reduced discrimination between social targets (Figure 10B). Across comparable exposure groups, differences between diets were evident, with high-fat diet animals generally showing reduced stranger preference relative to normal diet animals at higher Cr(VI) concentrations (Figure 10B,C). When considering these data as a ratio (cagemate/stranger), we observed 1) the ratio decreased after exposure to Cr(VI) in the normal diet group and 2) this effect was absent in the high-fat diet group (Supplementary Figure S2A). This ratio change was more pronounced in male rats than in female rats, though males also exhibited more variability than females (Supplementary Figure S2B,C). After 11 weeks, a similar diet-dependent pattern was observed. When sexes were pooled, high-fat diet control rats exhibited more interactions with stranger rats (purple bars) than normal diet controls, while cagemate interactions were similar between diets (Figure 10D). With increasing Cr(VI) exposure, changes were primarily observed with stranger interactions, whereas cagemate interactions remained relatively stable. At 0.1 mg/L Cr(VI), normal diet rats showed greater interactions with stranger rats than high-fat diet rats, indicating a clear difference between diets within the same exposure group. In contrast, cagemate interactions at this concentration were similar across diets, further indicating that diet-dependent effects were primarily associated with changes in stranger interactions (Figure 10D). Specifically, the average numbers of cagemate interactions with sexes pooled were 5.8, 8.3, and 5.8 for control, 0.05, and 0.1 mg/L Cr(VI) combined with normal-fat diet, and 7.1, 7.7, and 5.6 for high-fat diet control, 0.05, and 0.1 mg/L Cr(VI), respectively. The average numbers of stranger interactions for sexes pooled were 10.3, 11.1, and 13.4 for control, 0.05, and 0.1 mg/L Cr(VI) combined with normal-fat diet, and for high-fat diet groups were 13.7, 11.8, and 10.9 for control, 0.05, and 0.1 mg/L Cr(VI), respectively. We observed an overall similar pattern in the males, though we also noted an increase in cagemate interactions in the normal diet with 0.05 mg/L Cr(VI) group, which was the only group to exhibit more cagemate interactions than stranger interactions (Figure 10E). In both male and female rats fed a high-fat diet, Cr(VI) decreased interactions with stranger rats with a small decrease on cagemate interactions in 0.1 mg/L groups only (Figure 10E,F). These effects were consistent in males and females, where differences between diets were primarily driven by changes in stranger interactions rather than cagemate interactions (Figure 10E,F). When we consider these data as ratios for each rat, we observed the ratios increased after exposure to 0.05 mg/L Cr(VI) in both diet groups (Supplementary Figure S2D). This change was observed in male rats, while the ratios of female rats did not change significantly and only increased when comparing the high-fat diet to the normal diet (Supplementary Figure S2E,F).

### 3.6. High-Fat Diet Affected Cr Brain Accumulation

ICP-MS was utilized to quantify the accumulation of Cr in six isolated brain regions (striatum, hypothalamus, cerebellum, cortex, hippocampus, and brainstem; Figures 11 and 12). In control groups, Cr levels differed across brain regions and showed distinct patterns between diets. In normal-fat diet rats, Cr levels were highest in the cortex, followed by the striatum, cerebellum, hippocampus, hypothalamus, and brainstem. In contrast, high-fat diet rats showed the highest Cr levels in the striatum, followed by the cortex, cerebellum, hypothalamus, hippocampus, and brainstem, indicating diet altered the regional distribution of chromium in the brain (Figures 11 and 12).

In the normal-fat diet groups, Cr(VI) exposure produced limited increases in brain chromium levels. The only increase in brain Cr levels in the normal-fat diet groups was observed in the striatum of female rats exposed to 0.05 mg/L Cr(VI) (Figure 11C). Unexpectedly, exposure to Cr(VI) reduced Cr levels in the cerebellum of rats (Figure 11G–I). Similarly, we observed decreased Cr levels in the hypothalamus of normal-fat diet female rats exposed to 0.05 mg/L Cr(VI) and in the cortex of female rats exposed to 0.1 mg/L Cr(VI) (Figures 11F and 12C).

Interestingly, we observed Cr levels were significantly higher in high-fat diet relative to exposure-matched normal-fat diet groups in the striatum, hypothalamus, and cerebellum of rats (Figure 11). Specifically, mean Cr levels in the striatum when sexes were pooled were 70.1, 182.6, and 96.9 ng Cr/g tissue for control, 0.05, and 0.1 mg/L Cr(VI) combined with normal-fat diet, and 152.0, 200.5, and 129.4 ng Cr/g tissue for control, 0.05, and 0.1 mg/L Cr(VI) combined with high-fat diet, respectively. Mean Cr levels in the hypothalamus were 22.9, 19.8, and 19.7 ng Cr/g tissue for control, 0.05, and 0.1 mg/L Cr(VI) combined with normal-fat diet, and 31.8, 28.4, and 28.6 ng Cr/g tissue for sexes pooled control, 0.05, and 0.1 mg/L Cr(VI) combined with high-fat diet, respectively. Mean Cr levels in the cerebellum were 54.5, 31.0, and 23.0 ng Cr/g tissue for sexes pooled control, 0.05, and 0.1 mg/L Cr(VI) combined with normal-fat diet, and 49.6, 48.1, and 37.8 ng Cr/g tissue for control, 0.05, and 0.1 mg/L Cr(VI) combined with high-fat diet, respectively. When considering sexes separately, we observed Cr levels in the hypothalamus of male rats exposed to 0.1 mg/L Cr(VI) and female rats exposed to 0.05 mg/L Cr(VI) or 0.1 mg/L Cr(VI) were higher than Cr(VI)-matched groups with normal-fat diet (Figure 11E,F). Compared with normal-fat diet, Cr levels were significantly higher in the striatum of male rats exposed to 0.05 mg/L Cr(VI) (Figure 11B). Remarkably, the striatal Cr levels of female rats from high-fat diet controls were significantly higher than those of females from normal-fat diet controls (Figure 11C). We also discovered that while Cr levels in the cerebellum decreased with normal-fat diet consumption, the high-fat diet groups exhibited no differences across Cr(VI) groups but significantly increased Cr levels relative to the cerebellum of male rats fed normal-fat diet (Figure 11H), whereas Cr levels decreased in the cerebellum of female rats with both diets (Figure 11I). We observed no significant differences in cortical Cr levels across groups, but a notable decrease in females fed normal diet and exposed to 0.1 mg/L Cr(VI) (Figure 12C). Unexpectedly, Cr levels in the hippocampus of rats (pooled sexes) in the high-fat diet group after exposure to 0.1 mg/L Cr(VI) were notably decreased compared with the control high-fat diet group (Figure 12D), though comparisons within either sex lacked significant differences (Figure 12E,F). In the brainstem Cr levels were generally decreased across groups relative to sex- and diet-matched controls; we observed a notable decrease in male rats in the high-fat diet group exposed to 0.05 mg/L Cr(VI) relative to high-fat diet controls (Figure 12H) and in the brainstem Cr levels of female rats in the high-fat diet group relative to the normal-fat diet group after exposure to 0.1 mg/L Cr(VI) (Figure 12I).

### 3.7. Cr(VI) Exposure and High-Fat Diet Disrupt Essential Metal Homeostasis in Rat Brains

The homeostasis of essential metals, including iron (Fe), copper (Cu), selenium (Se), magnesium (Mg), cobalt (Co), manganese (Mn), and zinc (Zn), plays crucial roles in maintaining brain function, and deviations from normal levels can lead to neurological dysfunction [51–54]. We investigated the combined impact of Cr(VI) exposure and a high-fat diet on the concentrations of these essential metals in different brain regions of male and female rats, and the individual metal levels are shown in Supplementary Table S2. Here, we report relative fold-change in essential metals compared to sex- and diet-matched controls, then consider fold-change in all high-fat diet groups relative to Cr(VI)-matched normal diet groups (Figure 13).

Across regions, the magnitude of metal alterations varied substantially. The striatum exhibited the largest changes, with fold differences approaching ~8-fold for some metals. In contrast, intermediate changes of approximately ~4-fold or ~5-fold were observed in regions such as the hippocampus and brainstem, whereas other regions including the cerebellum and cortex generally showed smaller ~2-fold differences (Figure 13). These findings indicate that chromium exposure and diet produced region-specific effects with markedly different magnitudes, with the striatum showing the most pronounced alterations, followed by moderate changes in the hippocampus and brainstem, and comparatively smaller shifts in the cerebellum and cortex.

In the striatum, our findings revealed sex-specific alterations in metal levels following Cr exposure. We observed several opposite effects across sexes in the normal-fat diet groups, including on Fe, Se, Co, Mn, and Zn (Figure 13A). In the high-fat diet group, we observed opposite effects between sexes for Se; in females we observed opposite effects between diets for Fe and Mn. Moreover, when we normalize the fold-change of metals in high-fat diet groups relative to normal diet, we observed Se and Mn levels were significantly elevated in males, whereas in females Se and Zn levels were significantly reduced and Mn was significantly increased.

In the hypothalamus, our results demonstrated clear sex-specific alterations in essential metal levels following Cr(VI) exposure with a normal-fat diet (Figure 13B). In males fed a normal-fat diet, Cr exposure generally led to increased metal levels, whereas all essential metal levels were decreased in females. In the high-fat diet groups, Se levels were significantly elevated in males but reduced in females following Cr(VI) exposure. When normalizing high-fat diet levels relative to normal-fat diet groups, high-fat diet generally increased essential metal levels in both sexes, with the exception of unchanged Mg levels in female controls and males exposed to 0.1 mg/L and slight reductions in Mn in both sexes.

In the cerebellum, males in the normal-fat diet groups exposed to Cr(VI) led to a reduction in all essential metals except for those exposed to 0.05 mg/L that exhibited increased Mn, whereas female rats in the normal-fat diet groups exhibited mixed responses in essential metal levels (Figure 13C). In high-fat diet groups, we observed no significant changes in the levels of essential metals in either male or female rats; notably, Cr(VI) exposure caused a decrease in Co levels in females, whereas in males, Co levels showed a slight increase at 0.05 mg/L and a slight decrease at 0.1 mg/L. In contrast, Mn levels increased in males but decreased in females following Cr(VI) exposure. Normalizing the two diets, high-fat conditions led to an overall significant reduction in essential metals in males, whereas females only exhibited significant decreases in Cu and Mg. Notably, we observed opposite effects of high-fat diet on Co levels between sexes.

In the cortex, there were clear sex-specific changes in the levels of essential metal elements after Cr(VI) exposure (Figure 13D). Cr(VI) exposure in the normal-fat diet group resulted in increased essential metal levels in males, with Fe levels rising most prominently, while essential metal levels all decreased in females. We also observed opposite effects in males between diets, with the high-fat diet groups exhibiting decreased essential metals, particularly Mg, Mn, and Zn, whereas females fed high-fat diet exhibited similar effects (though attenuated) to females fed the normal-fat diet. When normalizing diets, we observed opposite effects between sexes for the control and 0.1 mg/L groups. High-fat diet led to an overall increase in trace metals in control males, whereas control female levels remained stable or decreased. After exposure to 0.05 mg/L Cr(VI), Fe, Mn, and Zn levels declined significantly in males, whereas female metal levels were not significantly altered. Exposure to 0.1 mg/L Cr(VI) induced minor reductions in male metal levels, but significantly increased essential metals in females.

In the hippocampus, Cr exposure under a normal-fat diet led to significantly increased Fe and Cu levels in males, whereas essential metals in females generally exhibited decreased levels (Figure 13E). Under high-fat diet conditions, essential metal levels in both male and female rats generally decreased, with females exhibiting significance for Mg and Co. Notably, we observed opposite effects between sexes for Mn levels, with females exhibiting decreased Mn following 0.1 mg/L Cr(VI) exposure and males displaying a slight increase (Figure 13E). Normalizing diets, male rats on a high-fat diet control exhibited no changes in essential metals, while females generally demonstrated reduced levels of essential metals, with the exception of Mn, which was significantly increased. Cr(VI) exposure generally resulted in decreased essential metals in both sexes, but we observed opposite effects for Mn with an increase in females at 0.05 mg/L Cr(VI) and in males at 0.1 mg/L Cr(VI) exposure.

In the brainstem, Cr exposure exhibited some opposite effects between sexes fed normal-fat diet; Cu was significantly increased in males exposed to 0.05 mg/L but decreased in females of the same Cr(VI) group, while Mg was significantly decreased in females exposed to 0.1 mg/L but slightly increased in males of the same Cr(VI) group. Males exhibited slightly elevated essential metals (Cu, Se, Mg), which were decreased in females; females only exhibited an increase in Mn, which was decreased in males. Under a high-fat diet, essential metals in males exhibited minor fluctuations, while levels in female rats mostly decreased, particularly Mg, Co, and Mn (Figure 13F). Notably, Mn levels increased slightly in males but significantly decreased in females after exposure to 0.1 mg/L. When normalizing diets, male control groups had generally unchanged levels except for a significant decrease in Fe, whereas female control groups had generally decreased levels, except for an increase in Mn. After Cr(VI) exposure, essential metals in males generally exhibited slight decreases, except for significant Mg and Zn reductions at 0.05 mg/L Cr(VI). In females, most essential metals slightly declined, except Mn increased at 0.05 mg/L Cr(VI). Mn levels rose slightly in males but declined in females after 0.1 mg/L Cr(VI) exposure.

## 4. Discussion

Both metal exposure and a high-fat diet are considered health risk factors that are particularly concerning for brain health. However, research on their combined effects remains limited, despite the growing urgency to understand their impact. Recent studies indicate co-exposure to a high-fat diet with lead or cadmium can lead to cognitive decline in animal models [55–58]. Expanding on this knowledge, we examined the effects of low-concentration Cr(VI) exposure combined with a high-fat diet on neurobehavioral outcomes, brain metal accumulation, and essential metal homeostasis. Interactions between dietary factors and metal toxicity have been reported previously for several environmental metals, including lead and cadmium. Similar to our observations, these studies have demonstrated that dietary status can modify metal toxicokinetics, tissue accumulation, and neurobehavioral outcomes. However, Cr(VI) possesses unique toxicological properties because it enters cells through anion transport systems and undergoes intracellular reduction to reactive intermediates that generate oxidative stress and DNA damage. Therefore, although our findings support the broader concept that high-fat diet can modify metal toxicity, the mechanisms underlying Cr(VI)-high-fat diet interactions may differ from those reported for other metals. To model realistic drinking water exposure, Cr(VI) was added to drinking water at 0.05 and 0.1 mg/L, concentrations corresponding to the EPA and WHO maximum contaminant levels for total Cr. This is especially concerning given that Cr(VI) often constitutes the majority of total Cr in drinking water (nearly 1:1 ratio), and a recent national survey estimated that 94% of the U.S. population is exposed to Cr(VI) at concentrations exceeding health-based reference thresholds [15,20].

We observed that Cr(VI) impaired grip strength in rats, while a high-fat diet attenuated this effect, especially in male rats. Metal exposure is reported to reduce grip strength in many studies [59–62]. Wu et al. (2022) [59] investigated the relationship between metal exposure and muscle strength in children and adolescents. The study found that metals such as cobalt, molybdenum, lead, antimony, strontium, thallium and cesium in urine, and their mixtures were significantly associated with decreased grip strength [59]. Similarly, our findings demonstrate that Cr(VI) exposure reduced grip strength in rats, supporting the broader association between metal exposure and impaired muscle function. The reduced grip strength in rats following Cr(VI) exposure may be attributed to muscle toxicity, as some studies have reported muscular atrophy after Cr(VI) exposure [63,64]. The effects of a high-fat diet (63.7% kcal fat) on muscle depend on duration and age [65]. A study found that a 12-week high-fat diet (60% kcal fat) can improve muscle fatigue resistance in 8-week-old male C57BL/6J mice [66]. This finding parallels our observation that high-fat diet attenuated the reduction in grip strength associated with Cr(VI) exposure, particularly in male rats. However, another study found that a 5-week high-fat diet (45% kcal fat) did not change grip strength in 6-week-old C57BL/6 mice [67]. Interestingly, our study found that a high-fat diet could alleviate Cr(VI)-impaired grip strength in rats. While diet-dependent modification of metal toxicity has been reported for other metals, including lead and cadmium, similar interactions have not been well characterized for Cr(VI).

Co-exposure to Cr(VI) and a high-fat diet reduced locomotor activity in rats, suggesting impaired motor function and/or motivation. Similar reductions in activity have been reported following Cr(VI) exposure and other neurotoxic metal exposures, indicating that locomotor deficits may represent a sensitive endpoint of metal-induced neurotoxicity. Cr(VI) has been reported to induce neurotoxic effects, including oxidative stress, neuroinflammation, and neuronal damage, which can impair neural function and behavior [5]. Sánchez-Díaz et al. investigated metal pollution in Spanish rivers associated with motor neuron disease deaths in humans and found Cr(VI) increased the risk of motor neuron disease [21]. Cr(VI) was also reported to reduce locomotor activity in experimental animals in some previous studies [26,27,68]. Consistent with these reports, we observed reduced locomotor activity in rats exposed to Cr(VI), with the strongest effects occurring during co-exposure with a high-fat diet, as demonstrated by decreased distance traveled in the open-field assay and fewer arm entries in the Y-maze. A high-fat diet has also been shown to alter locomotor activity, with some studies reporting increased activity or hyperactivity during early stages of exposure [69–71], while others demonstrate high-fat diet can impair motor function through multiple mechanisms, including neuroinflammation, neuronal damage, and disruptions in dopamine pathways [72–74]. These effects may involve damage to brain regions that regulate motor and motivational behaviors, including the cerebellum and hypothalamus [72], as well as enhanced neuroinflammatory and cell death pathways under combined toxic stress, such as acrylamide [74]. In our study, co-exposure to Cr(VI) attenuated potential activity-promoting effects of the high-fat diet, resulting in an overall reduction in locomotor activity, as indicated by decreased distance traveled in the open-field assay and reduced arm entries in the Y-maze. Similar interactions have been observed with other environmental metals, such as lead, where co-exposure with a high-fat diet exacerbates neurobehavioral deficits and reduces activity levels [57]. Together, these findings suggest that combined dietary and environmental exposures produce additive or potentially synergistic neurotoxic effects, leading to impaired locomotor activity. These findings extend previous observations of diet–metal interactions and suggest that co-exposure to Cr(VI) and a high-fat diet can alter locomotor activity, highlighting the interactive effects of dietary and environmental exposures on behavioral outcomes.

The effects of Cr(VI) on anxiety-like behavior depended strongly on dietary condition. Under normal-fat diet conditions, Cr(VI) exposure tended to reduce anxiety-like behavior, whereas co-exposure with a high-fat diet generally shifted responses toward increased anxiety-like behavior. These findings suggest that dietary status may substantially modify the neurobehavioral consequences of Cr(VI) exposure. Our previous study also reported long-term Cr(VI) exposure induced anxiety in 7-month-old and 18-month-old rats in these same assays [28,29]. Boopathi et al. found that exposure to Cr(VI) increased anxiety-like behavior in zebrafish in the open-field test [75]. A high-fat diet can significantly increase anxiety levels [76,77]. Li et al. found a high-fat diet impaired autophagy by decreasing AMPK phosphorylation and increasing mTOR phosphorylation, which in turn contributed to anxiety-like behavior in 7–8-week-old male C57BL/6 mice, using the open-field test and elevated plus maze [77]. In our study, while a high-fat diet alone did not increase anxiety in rats, it unexpectedly led to anxiety-like behavior in males after three weeks, as indicated by increased open-arm exploration in the elevated plus maze. Notably, co-exposure to Cr(VI) and a high-fat diet shifted this response toward increased anxiety-like behavior, as reflected by reduced open-arm exploration (and decreased center exploration in the open-field assay), suggesting an interaction between dietary and environmental factors. Similarly, Iqbal and Ahmed reported that co-exposure to a mixture of metals (aluminum, copper, lead, and cadmium) and a high-fat diet increased anxiety levels in young mice, as measured by the elevated plus maze [43]. Limited information is available regarding the effects of combined Cr(VI) exposure and high-fat diet on anxiety-like behavior, and our findings contribute new evidence that dietary status can modify Cr(VI)-associated behavioral responses.

Co-exposure to Cr(VI) and a high-fat diet produced relatively modest effects on spatial memory, with evidence of transient impairment primarily in females. The disappearance of these effects at later time points suggests the possibility of adaptive or compensatory responses following prolonged exposure. The impact of a high-fat diet on spatial memory has been widely investigated, particularly regarding hippocampal function, with most evidence showing that it impairs spatial memory and hippocampus-dependent cognitive performance in rodents [78–80]. However, in the present study, spatial memory remained largely unchanged following prolonged co-exposure, which contrasts with a previous study showing that a high-fat diet improves working memory in male C57BL/6J mice using the Y-maze test [81]. Overall, these findings indicate that Cr(VI) and high-fat diet co-exposure induced a transient, female-specific impairment in spatial working memory, which diminishes with prolonged exposure, suggesting the involvement of adaptive or compensatory mechanisms.

Social behavior was particularly sensitive to the combined effects of Cr(VI) and high-fat diet. Although overall sociability was largely preserved, social novelty preference was consistently altered, especially in females. These findings suggest that combined exposure may preferentially disrupt social recognition and discrimination rather than general social interaction. Although the increased preference for social novelty in rodents after exposure to neurotoxic substances has not been widely demonstrated, our previous study reported elderly rats also exhibited increased preference for social novelty after Cr(VI) exposure [28,29]. Similarly, young rats in the present study showed altered social novelty preference following Cr(VI) exposure, suggesting that changes in responses to novel social stimuli may represent a reproducible behavioral effect of Cr(VI) across different age groups. While high-fat diet has been reported to reduce sociability in mice, as measured by decreased time spent in the social chamber and reduced proximity to conspecifics in the 3-chamber test [82]. In our study, a high-fat diet alone did not reduce the social novelty preference of rats, but co-exposure with Cr(VI) significantly reduced the interaction of rats with stranger rats. Together, these findings indicate that co-exposure to Cr(VI) and a high-fat diet disrupts social behavior by altering social preference and recognition, rather than uniformly reducing sociability, and that these effects are driven primarily by changes in interactions with novel social stimuli. Our findings provide new evidence that combined Cr(VI) exposure and high-fat diet can alter social behavior and social novelty preference in rats, extending the growing literature describing interactions between dietary factors and environmental metal exposures.

Brain Cr accumulation was strongly influenced by both brain region and dietary status. High-fat diet promoted Cr accumulation in several regions, particularly the striatum and hypothalamus, suggesting that dietary factors can substantially modify Cr toxicokinetics within the central nervous system. Few studies have reported brain Cr levels in rodents following drinking water exposure, and data on specific brain regions are particularly limited. One group measured Cr levels in the hypothalamus and pituitary of adult male Wistar rats after 30 days of exposure to 100 or 500 ppm potassium dichromate using ICP-MS, reporting pituitary levels of 274 and 640 ng/g, and hypothalamus levels of 26 and 59 ng/g, respectively [23,83]. In a previous study, we observed selective Cr accumulation in the hippocampus of middle-aged and elderly female rats after 90 days of exposure to Cr(VI) in drinking water at concentrations of 0.05 or 0.1 mg/L [24]. Considering that the rats in this study were adolescents, age may contribute to differences in regional Cr deposition in the brain. A high-fat diet was reported to influence metal levels in tissues such as the liver, kidney, heart, testis, and brain [46,84–86]. For instance, Young et al. (2019) [84] reported mice exposed to environmentally relevant concentrations of cadmium throughout their lifetime and maintained on a high-fat diet exhibited higher cadmium levels in the kidney, liver, and heart compared to those on a low-fat diet. Similarly, Liu et al. found that high-fat diet-fed mice exhibited higher accumulation of metals such as arsenic, lead, and cadmium in the liver and kidney compared with mice fed a normal diet [85]. Mazzocco et al. also demonstrated high-fat diet led to increased cadmium accumulation in the brains of mice exposed to cadmium throughout their lifespan [46]. More recently, Zhang et al. showed that in C57BL/6 male mice, a high-fat diet elevated cadmium levels in the hippocampus after 10 weeks of cadmium exposure [55]. However, in another study of four-week-old male Sprague Dawley rats exposed to lead and a high-fat diet for 12 weeks, the high-fat diet was not found to increase brain lead levels compared with a standard diet [57]. Our study demonstrates that high-fat diet promotes Cr accumulation in specific brain regions. While similar diet-dependent increases in tissue accumulation have been reported for cadmium and other metals, evidence for such effects with Cr(VI) has been limited. Notably, we observed high-fat diet increased Cr levels in the striatum and hypothalamus, which are brain regions that play critical roles in motor control, balance, coordination, and emotional regulation, among other behaviors. By highlighting the combined effects of high-fat diet and Cr(VI) exposure on brain Cr distribution, our study addresses a significant gap in understanding how dietary factors modulate the neurotoxicity risk of Cr(VI).

Combined Cr(VI) exposure and high-fat diet disrupted essential metal homeostasis in a region-specific manner, supporting the hypothesis that dietary and environmental factors interact to alter metallomic regulation in the brain. Notably, elements such as Mn and Se showed particularly dynamic changes across multiple regions, suggesting that they may be especially sensitive to combined metabolic and toxicological stress. Essential metal homeostasis is crucial for the proper functioning of the nervous system [87]. Essential metals like Cu, Fe, Mn, and Zn are crucial cofactors in cellular processes such as enzymatic activity, mitochondrial function, and neurotransmission, playing a key role in the development and function of the nervous system, including neurotransmission and myelination [87–89]. Essential metal dyshomeostasis can contribute to neurological disorders and neurodegenerative diseases, with imbalances in metal homeostasis associated with conditions like autism spectrum disorder, Alzheimer’s disease, Parkinson’s disease, and amyotrophic lateral sclerosis [90]. Disorders in Cu and Fe maintenance, such as Menkes and Wilson diseases, further emphasize the critical role these metals play in preventing neurodegeneration [51]. Metal exposure, such as cadmium exposure, can disrupt the homeostasis of essential metals in the body [84]. Recent studies have demonstrated that cadmium exposure disrupts the homeostasis of essential metals in organs such as the liver, kidney, heart, testes, and brain [46,84,86]. Similarly, our previous study observed low-dose Cr(VI) exposure through drinking water for 90 days induced disturbances in metal homeostasis in the hippocampus of rats [24]. Importantly, high-fat diet can aggravate the essential metal dyshomeostasis caused by metal exposure [46,84,86]. Similarly, we observed that co-exposure to Cr(VI) and high-fat diet produced broader alterations in brain metal homeostasis than either factor alone, particularly affecting Mn and Se concentrations across multiple brain regions.

Sex differences are increasingly recognized as critical factors influencing susceptibility to metal-induced diseases, particularly neurological effects [91,92]. Our study found sex substantially influenced the behavioral and metallomic responses to combined Cr(VI) and high-fat diet exposure. Rather than exhibiting a uniform pattern of sensitivity, males and females displayed endpoint-specific responses, with females generally showing greater susceptibility in locomotor, anxiety-related, and social behavioral domains. Similar sex-dependent effects have been reported previously for both Cr(VI) exposure and high-fat diet models, supporting the importance of considering sex as a biological variable when evaluating neurotoxic outcomes. In one study of Cr(VI)-induced neurodevelopmental effects, a ten-fold increase in urinary Cr(VI) levels was associated with lower intelligence quotient scores in boys only [93]. Our previous work revealed clear sex-specific behavioral effects of Cr in middle-aged rats: Cr(VI) exposure led to a greater decline in motor function in females than in males, impaired spatial memory in females but improved it in males, and significantly reduced social ability in middle-aged males [28,29]. We also observed Cr accumulation in the brain of middle-aged and geriatric rats and the imbalance in metal homeostasis associated with Cr accumulation exhibited a sex-specific pattern, but Cr accumulated selectively in the hippocampus in middle-aged and geriatric females [24]. Similarly, some studies reported sex differences in the effects of a high-fat diet on the brain. A study reported female mice on a high-fat diet showed reduced adult hippocampal neurogenesis, which is linked to cognitive decline and mood disorders, while males did not exhibit such changes [94]. In contrast, another study found male mice exposed to high-fat diet displayed more pronounced alterations in sensorimotor integration and anxiety-like behavior (as indicated by reduced time spent in the center zone of the open-field test and decreased time in the light compartment of the light/dark box test) earlier than females [95]. To our knowledge, few studies have examined sex-dependent responses to combined Cr(VI) exposure and high-fat diet. Our findings provide new evidence that sex substantially modifies behavioral and neurobiological responses to this combined exposure paradigm. Our findings highlight the importance of considering sex as a biological variable in toxicological studies, especially when evaluating the complex interactions between environmental toxicants and diet.

Several limitations should be considered when interpreting these findings. First, measures of center exploration in the open-field assay may be influenced by concurrent changes in locomotor activity; therefore, conclusions regarding anxiety-like behavior are primarily supported by the elevated plus maze results. Second, although males and females were analyzed separately, formal statistical comparisons between sexes were not performed. Consequently, sex-specific differences should be interpreted cautiously and confirmed in future studies specifically designed to evaluate sex effects. Finally, this study focused on neurobehavioral outcomes and brain metal accumulation and did not directly investigate the molecular mechanisms underlying the observed interactions between Cr(VI) exposure and high-fat diet. Therefore, mechanistic explanations discussed above should be considered potential hypotheses requiring experimental validation.

## 5. Conclusions

Our study is the first to comprehensively investigate the combined effects of Cr(VI) exposure and high-fat diet on neurobehavioral function, metal accumulation, and essential metal homeostasis in the brain, while also examining sex-specific responses. We observed co-exposure to Cr(VI) and high-fat diet produced complex and often interactive effects on motor function, anxiety-like behaviors, social interactions, and brain metal distribution. Notably, high-fat diet mitigated Cr(VI)-induced grip strength deficits but exacerbated anxiety and impaired social novelty preference—effects that were sex-specific and suggest specificity to distinct brain regions. Furthermore, high-fat diet promoted Cr accumulation in key brain regions involved in motor control and emotional regulation, such as the striatum and hypothalamus, and altered essential metal homeostasis in a region- and sex-specific manner. These findings underscore the importance of considering both dietary factors and biological sex when evaluating the neurotoxic risks of environmental metals, and they provide valuable insights into the mechanisms underlying diet–metal interactions in the brain.

## Supplementary Material

supplementary data

**Supplementary Materials:** The following supporting information can be downloaded at: https://www.mdpi.com/article/10.3390/app16157367/s1, Figure S1: Cr(VI) and high-fat diet co-exposure impacted sociability in rats. Figure S2: Cr(VI) and high-fat diet co-exposure impacted the social novelty ratio in rats. Table S1: Two-way ANOVA results for behavioral outcomes. Table S2: Cr(VI) and high-fat diet co-exposure altered essential metal levels in the rat brain.

## Figures and Tables

**Figure 1.**
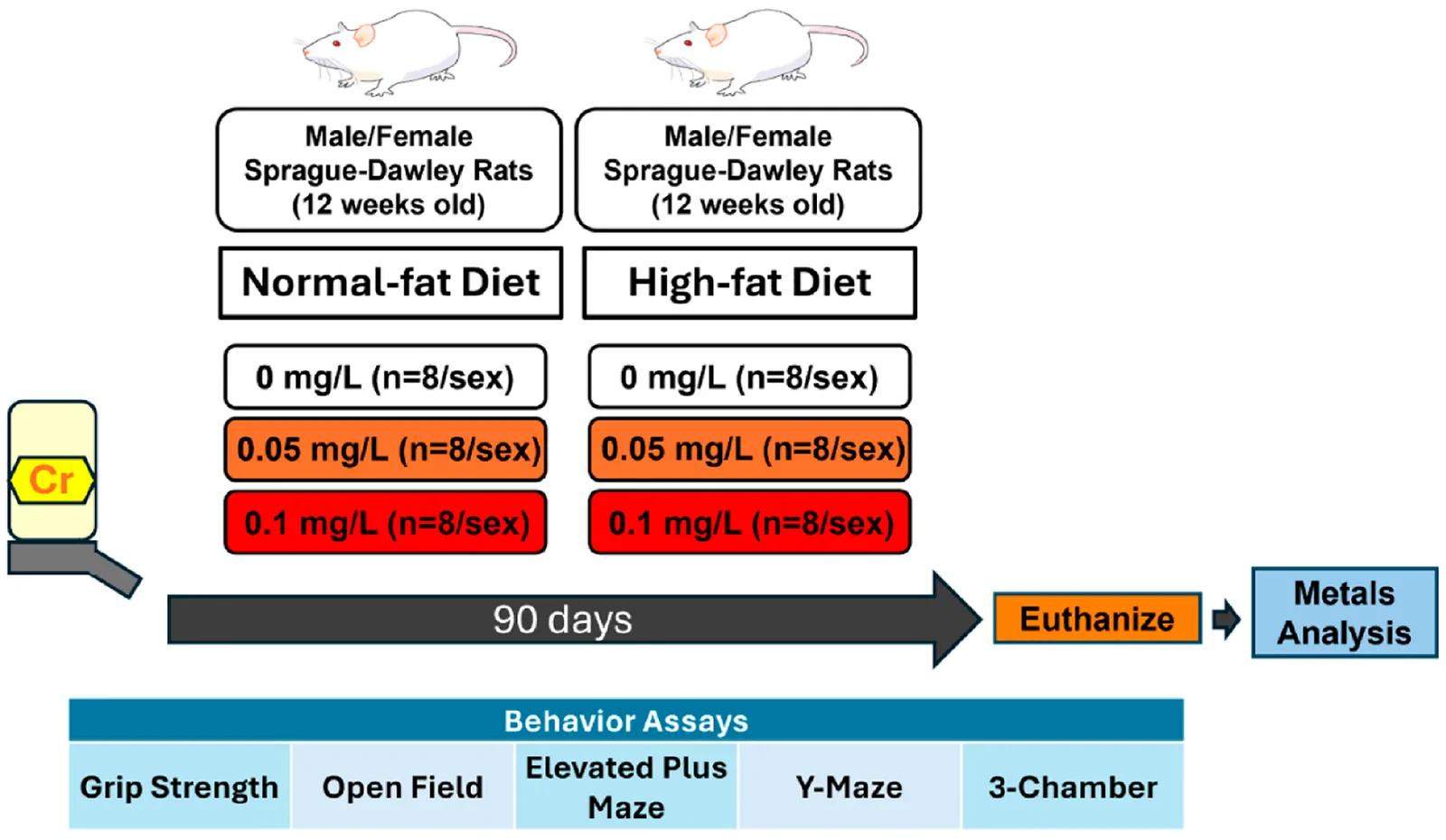
Rat model to assess the neurotoxicity of Cr(VI) and high-fat diet co-exposure. We exposed 48 male and 48 female rats to 0, 0.05, or 0.1 mg Cr(VI)/L for 90 days. We considered two diet groups to compare across normal-fat diet and high-fat diet rats. Throughout the 90-day exposure period, a series of behavioral tests were conducted to assess grip strength, motor function, anxiety, spatial memory, mobility, and social memory. After 90 days of exposure, rats were euthanized. Brains were isolated and then microdissected for metals analysis.

**Figure 2.**
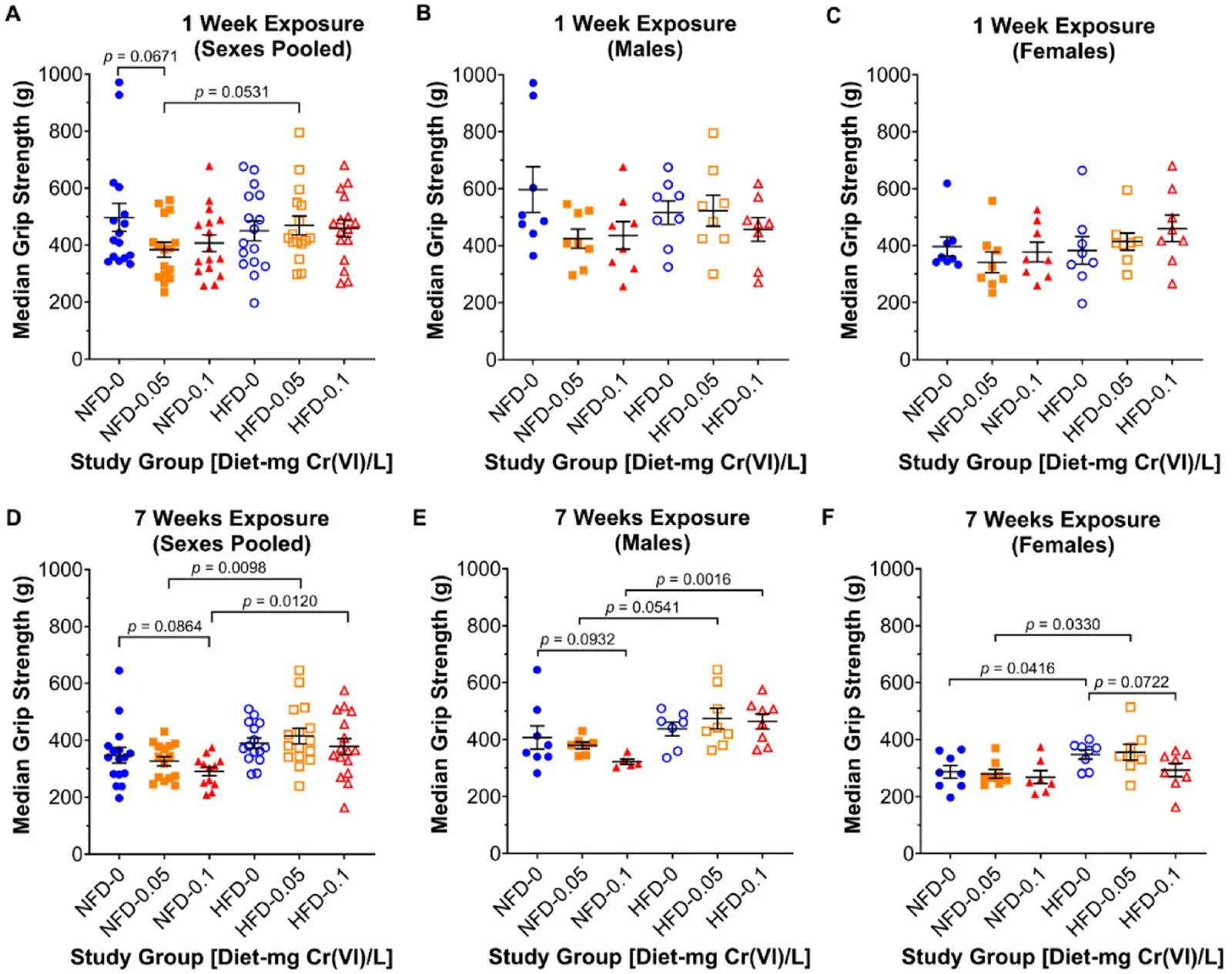
Cr(VI) reduced grip strength in rats fed a normal-fat diet, which was largely rescued by high-fat diet. We investigated the effects of co-exposure to Cr(VI) in drinking water with a high-fat diet for 1 or 7 weeks on grip strength in rats. (**A**) Effects of 1-week exposure on grip strength in all rats. (**B**) Effects of 1-week exposure on grip strength in male rats. (**C**) Effects of 1-week exposure on grip strength in female rats. (**D**) Effects of 7-week exposure on grip strength in all rats. (**E**) Effects of 7-week exposure on grip strength in male rats. (**F**) Effects of 7-week exposure on grip strength in female rats. Data points represent individual rats, bars represent mean ± SEM. *n* = 8 animals per sex per treatment group unless otherwise indicated. Missing data points are due to rats refusing to grasp the horizontal bar during the grip strength test. For week 7 analyses, sample sizes were reduced in the NFD + 0.1 mg/L Cr(VI) groups (male *n* = 5, female *n* = 7). Blue circles are control rats, orange squares are 0.05 mg Cr(VI)/L, and red triangles are 0.1 mg Cr(VI)/L; closed data points are normal-fat diet (NFD), and open data points are high-fat diet (HFD). The *p*-values of statistical differences are reported in the graphs.

**Figure 3.**
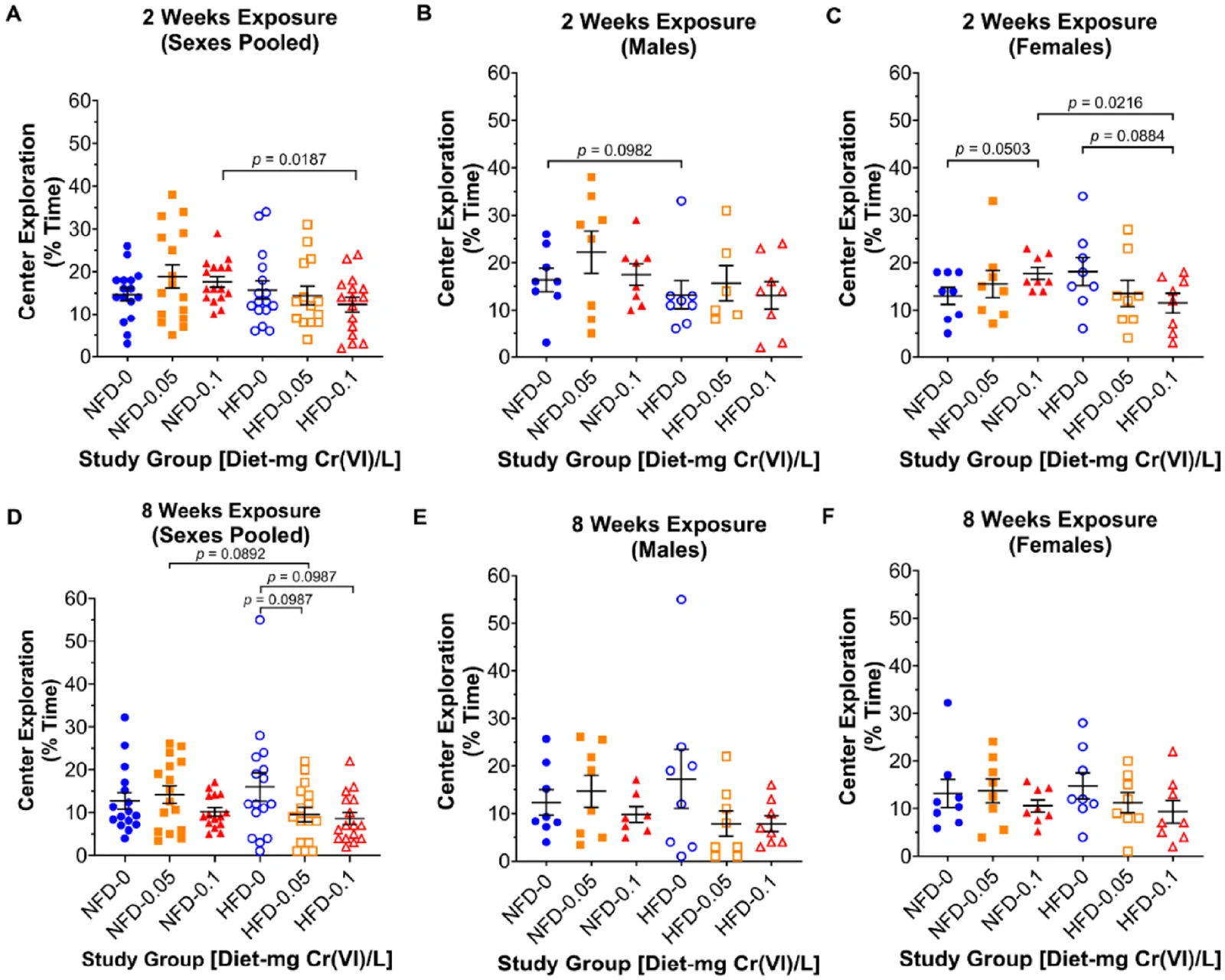
Cr(VI) and high-fat diet co-exposure decreased center area exploration in rats. We investigated the effects of co-exposure to Cr(VI) in drinking water with a high-fat diet for 2 or 8 weeks on center area exploration in rats. (**A**) Effects of 2-week exposure on center area exploration in all rats. (**B**) Effects of 2-week exposure on center area exploration in male rats. (**C**) Effects of 2-week exposure on center area exploration in female rats. (**D**) Effects of 8-week exposure on center area exploration in all rats. (**E**) Effects of 8-week exposure on center area exploration in male rats. (**F**) Effects of 8-week exposure on center area exploration in female rats. Data points represent individual rats, bars represent mean ± SEM. *n* = 8 animals per sex per treatment group unless otherwise indicated. For week 2 center exploration, the HFD + 0.05 mg/L Cr(VI) male group contained *n* = 6 animals. One outlier (47.73%) was removed from the 0.1 mg/L Cr(VI) with normal-fat diet male group after 8 weeks, resulting in *n* = 7 animals for that group. Blue circles are control rats, orange squares are 0.05 mg Cr(VI)/L, and red triangles are 0.1 mg Cr(VI)/L; closed data points are normal-fat diet (NFD) and open data points are high-fat diet (HFD). The *p*-values of statistical differences are reported in the graphs.

**Figure 4.**
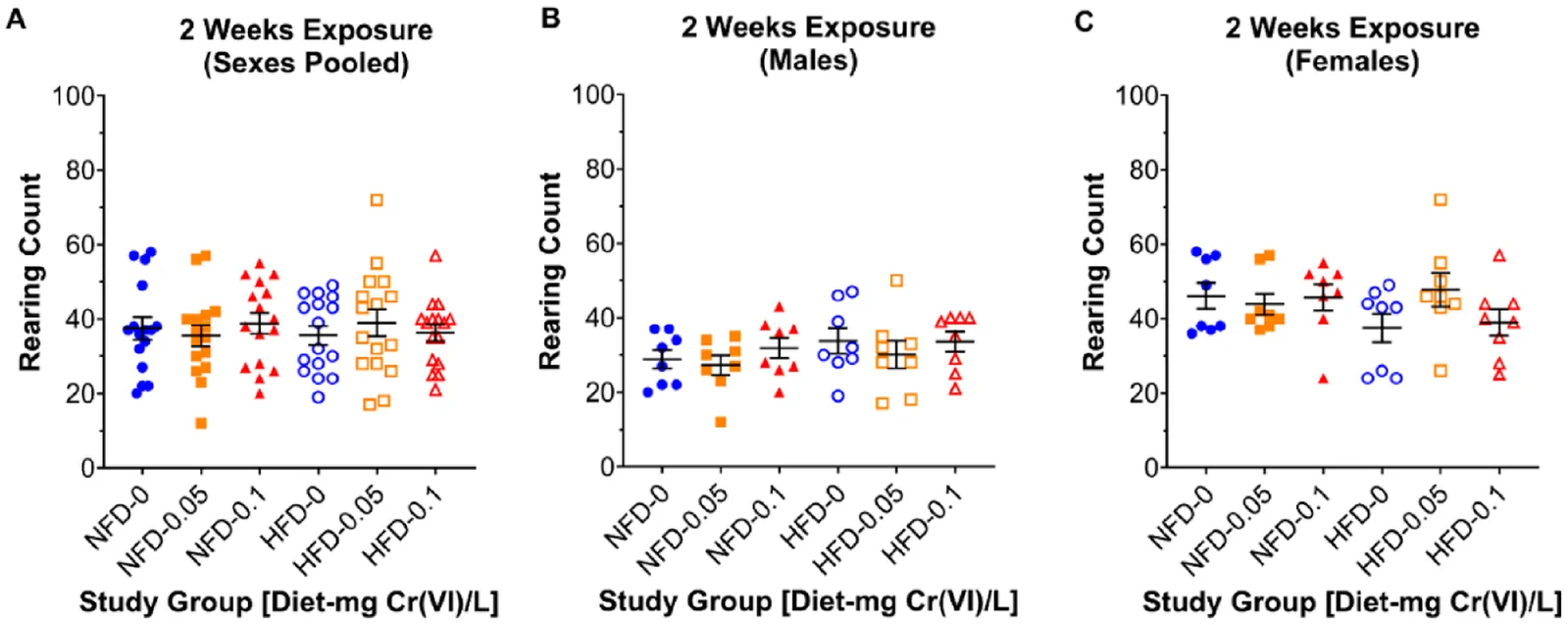

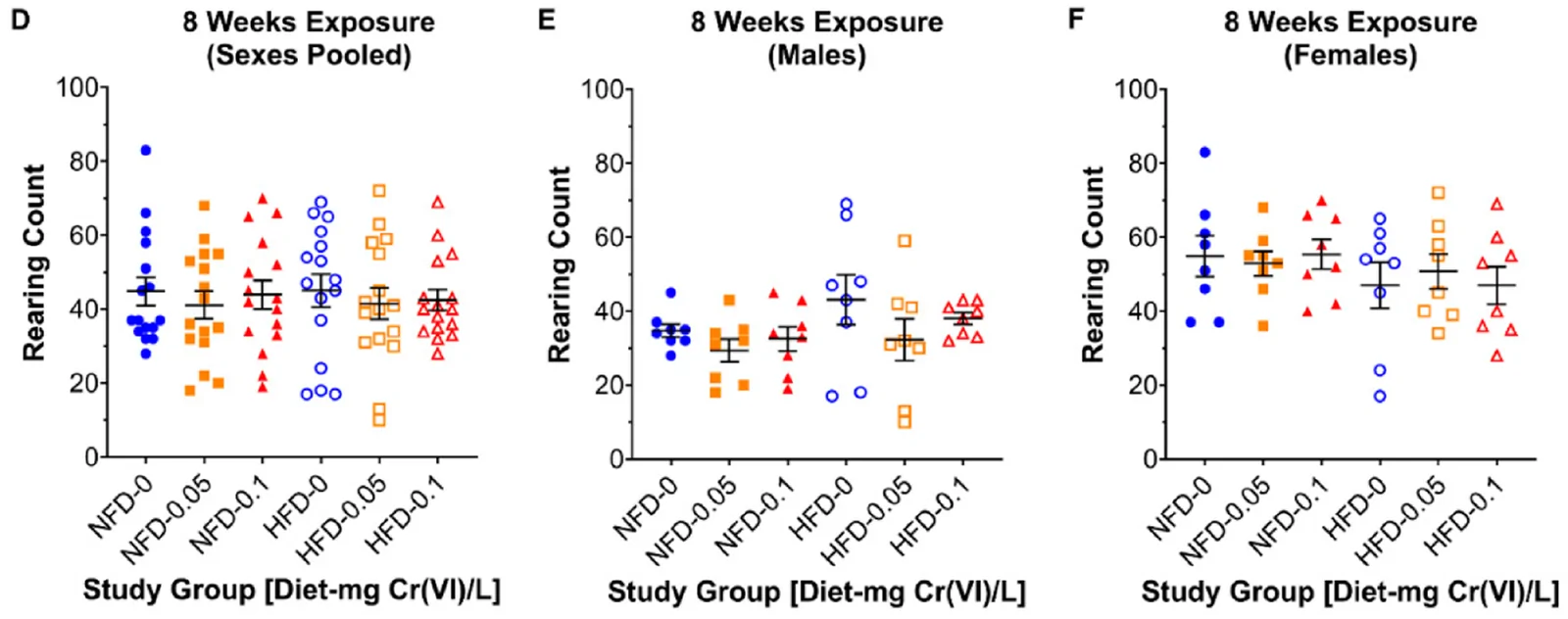
Cr(VI) and high-fat diet co-exposure did not impact rearing behavior in rats. We investigated the effects of co-exposure to Cr(VI) in drinking water and a high-fat diet for 2 or 8 weeks on rearing behavior in rats. (**A**) Effects of 2-week exposure on rearing behavior in all rats. (**B**) Effects of 2-week exposure on rearing behavior in male rats. (**C**) Effects of 2-week exposure on rearing behavior in female rats. (**D**) Effects of 8-week exposure on rearing behavior in all rats. (**E**) Effects of 8-week exposure on rearing behavior in male rats. (**F**) Effects of 8-week exposure on rearing behavior in female rats. Data points represent individual rats, bars represent mean ± SEM. *n* = 8 animals per sex per treatment group for all analyses. Blue circles are control rats, orange squares are 0.05 mg Cr(VI)/L, and red triangles are 0.1 mg Cr(VI)/L; closed data points are normal-fat diet (NFD), and open data points are high-fat diet (HFD).

**Figure 5.**
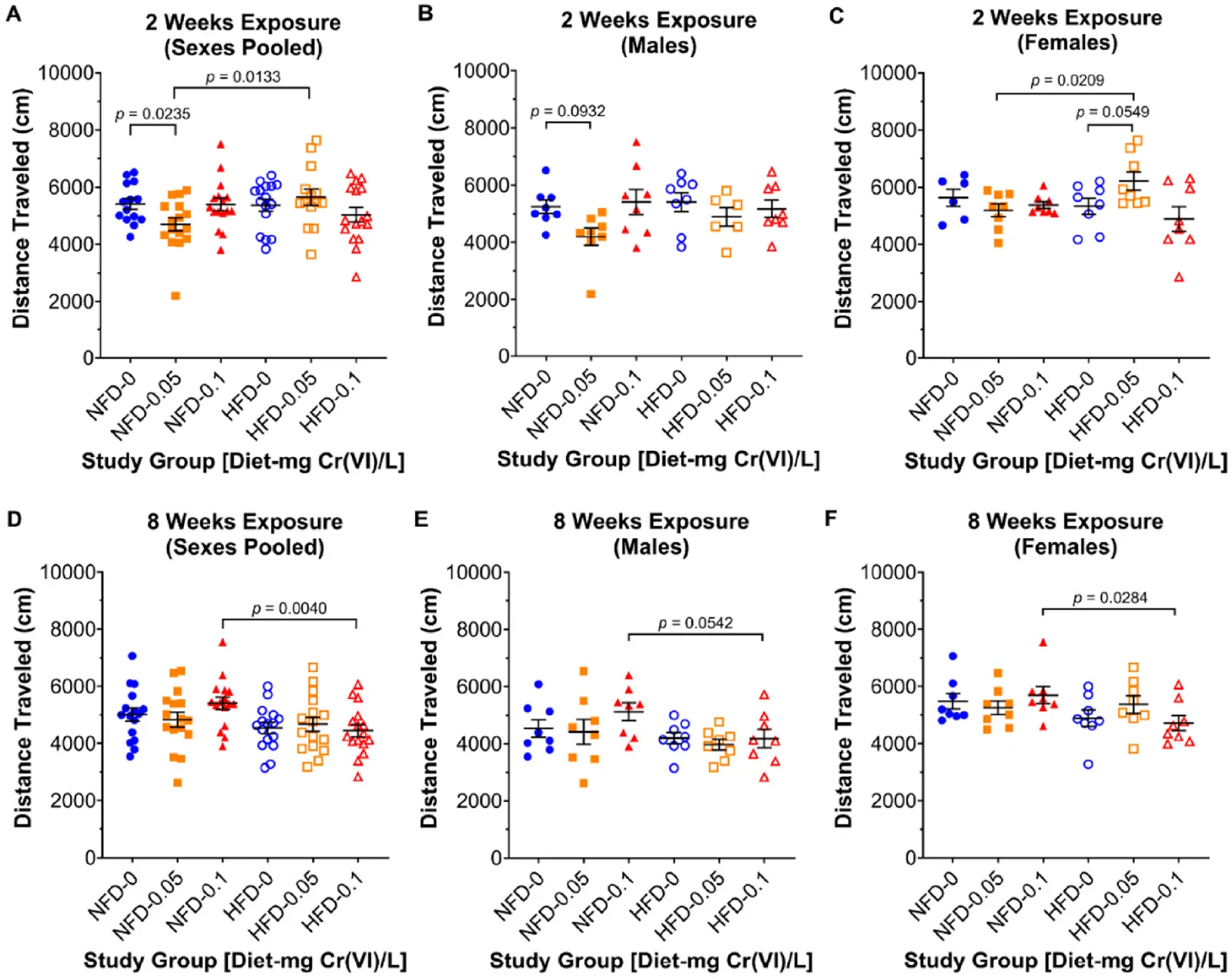
Cr(VI) and high-fat diet co-exposure impacted travel distance in rats. We investigated the effects of co-exposure to Cr(VI) in drinking water with a high-fat diet for 2 or 8 weeks on distance traveled during the open field test. (**A**) Effects of 2-week exposure on distance traveled in all rats. (**B**) Effects of 2-week exposure on distance traveled in male rats. (**C**) Effects of 2-week exposure on distance traveled in female rats. (**D**) Effects of 8-week exposure on distance traveled in all rats. (**E**) Effects of 8-week exposure on distance traveled in male rats. (**F**) Effects of 8-week exposure on distance traveled in female rats. Data points represent individual rats, and bars represent mean ± SEM. Two outliers (9509.8 cm and 12,880.7 cm) were removed from the normal-fat diet alone female group after 2 weeks, resulting in *n* = 6 animals for that group. The HFD + 0.05 mg/L Cr(VI) male group at week 2 also contained *n* = 6 animals. All other groups contained *n* = 8 animals per sex per treatment group. Blue circles are control rats, orange squares are 0.05 mg Cr(VI)/L, and red triangles are 0.1 mg Cr(VI)/L; closed data points are normal-fat diet (NFD), and open data points are high-fat diet (HFD). The *p*-values of statistical differences are reported in the graphs.

**Figure 6.**
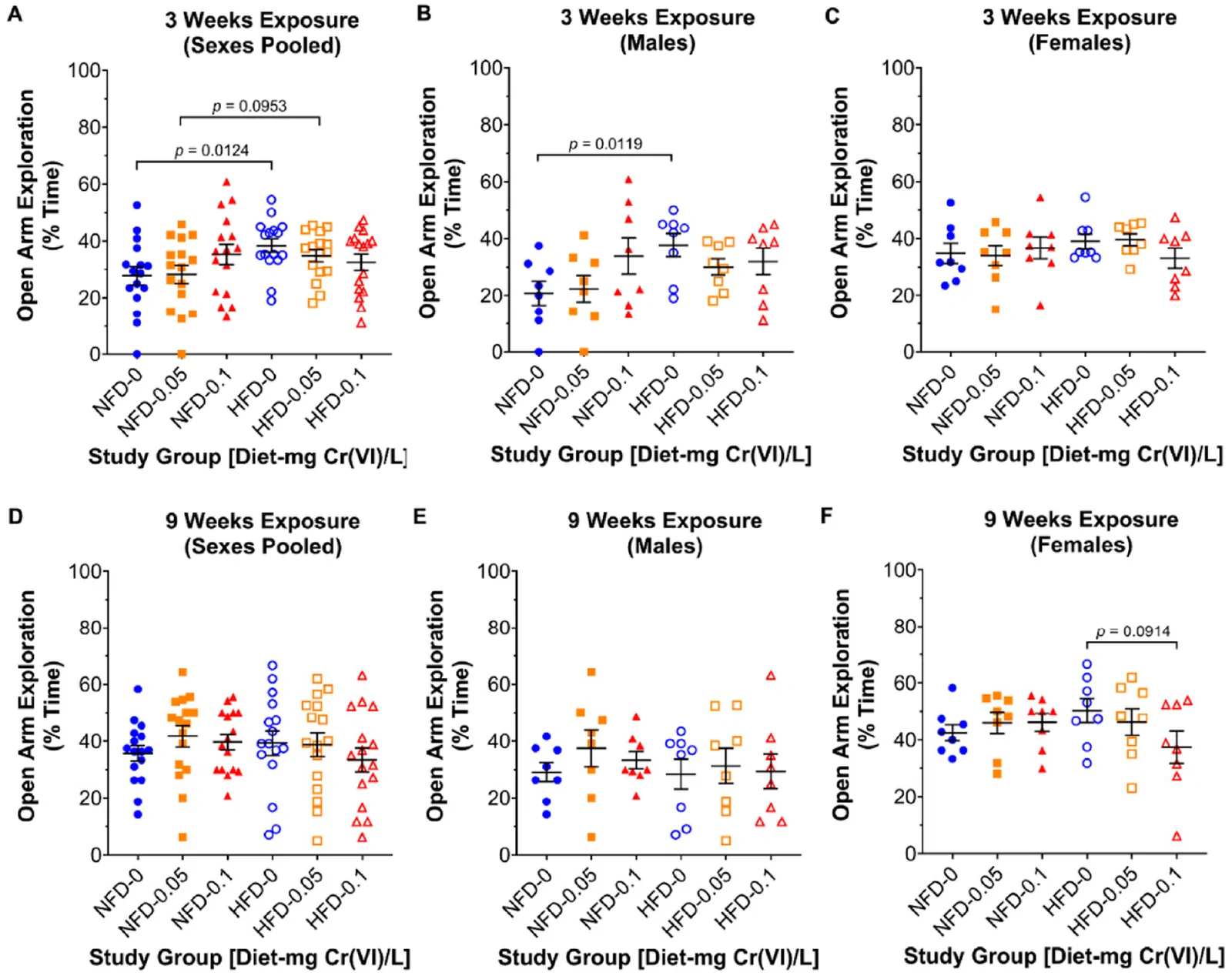
Cr(VI) and high-fat diet co-exposure impacted anxiety in rats. We utilized the elevated plus maze to investigate the effects of co-exposure with Cr(VI) in drinking water and high-fat diet for 3 or 9 weeks on open arm exploration in rats. (**A**) Effects of 3-week exposure on open arm exploration in all rats. (**B**) Effects of 3-week exposure on open arm exploration in male rats. (**C**) Effects of 3-week exposure on open arm exploration in female rats. (**D**) Effects of 9-week exposure on open arm exploration in all rats. (**E**) Effects of 9-week exposure on open arm exploration in male rats. (**F**) Effects of 9-week exposure on open arm exploration in female rats. Data points represent individual rats, and bars represent mean ± SEM. *n* = 8 animals per sex per treatment group for all analyses. Blue circles are control rats, orange squares are 0.05 mg Cr(VI)/L, and red triangles are 0.1 mg Cr(VI)/L; closed data points are normal-fat diet (NFD), and open data points are high-fat diet (HFD). The *p*-values of statistical differences are reported in the graphs.

**Figure 7.**
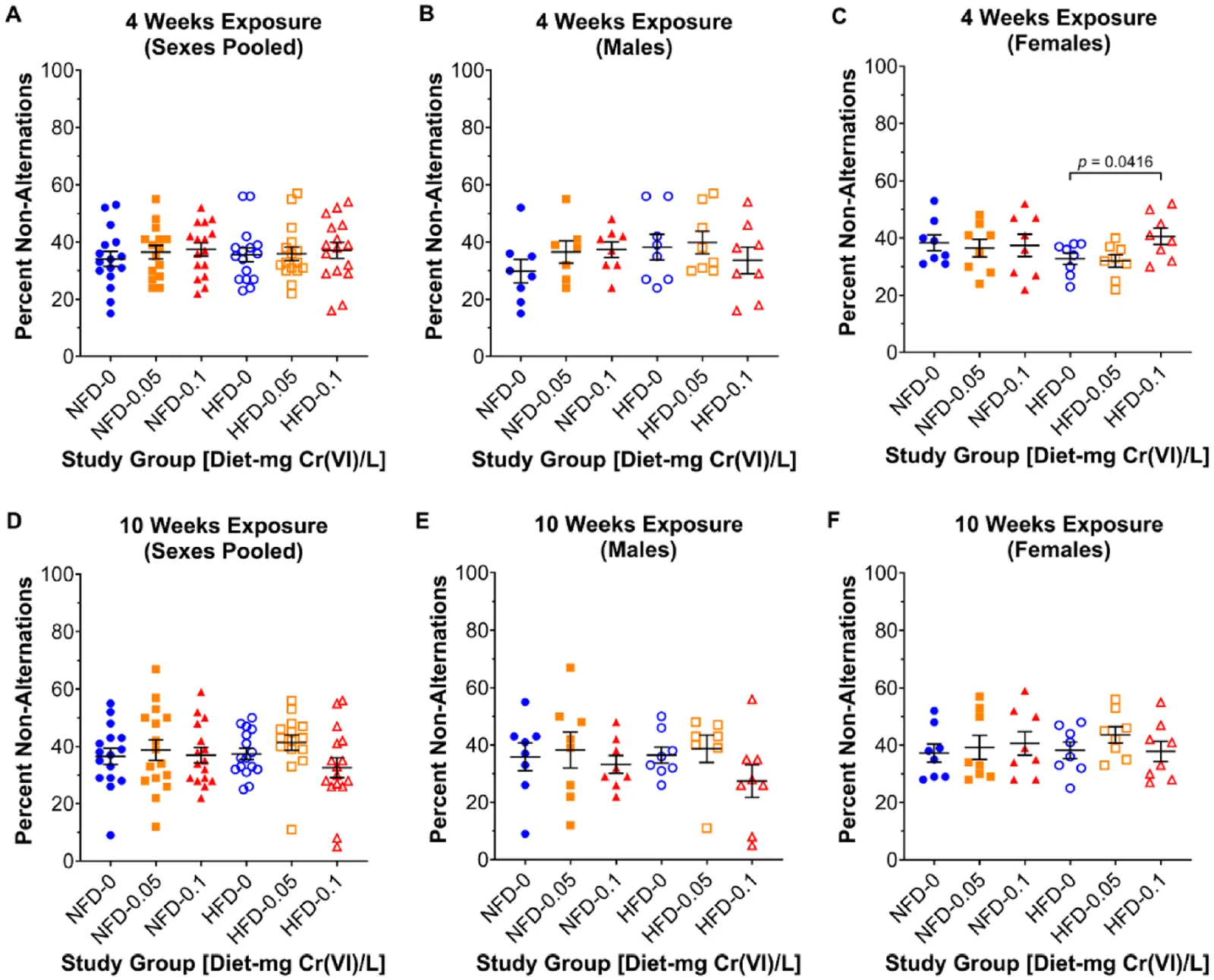
Cr(VI) and high-fat diet co-exposure impacted spatial memory in female rats more than males. We investigated the effects of co-exposure to Cr(VI) in drinking water and a high-fat diet for 4 or 10 weeks on non-alternating exploration in rats. (**A**) Effects of 4-week exposure on non-alternations in all rats. (**B**) Effects of 4-week exposure on non-alternation exploration in male rats. (**C**) Effects of 4-week exposure on non-alternations in female rats. (**D**) Effects of 10-week exposure on non-alternations in all rats. (**E**) Effects of 10-week exposure on non-alternations in male rats. (**F**) Effects of 10-week exposure on non-alternations in female rats. Data points represent individual rats, and bars represent mean ± SEM. One outlier (100%) was removed from the 0.05 mg/L Cr(VI) with normal-fat diet male group after 4 weeks, resulting in *n* = 7 animals for that group. One outlier (90%) was removed from the 0.05 mg/L Cr(VI) with high-fat diet male group after 10 weeks, resulting in *n* = 7 animals for that group. All other groups contained *n* = 8 animals per sex per treatment group. Blue circles are control rats, orange squares are 0.05 mg Cr(VI)/L, and red triangles are 0.1 mg Cr(VI)/L; closed data points are normal-fat diet (NFD), and open data points are high-fat diet (HFD). The *p*-values of statistical differences are reported in the graphs.

**Figure 8.**
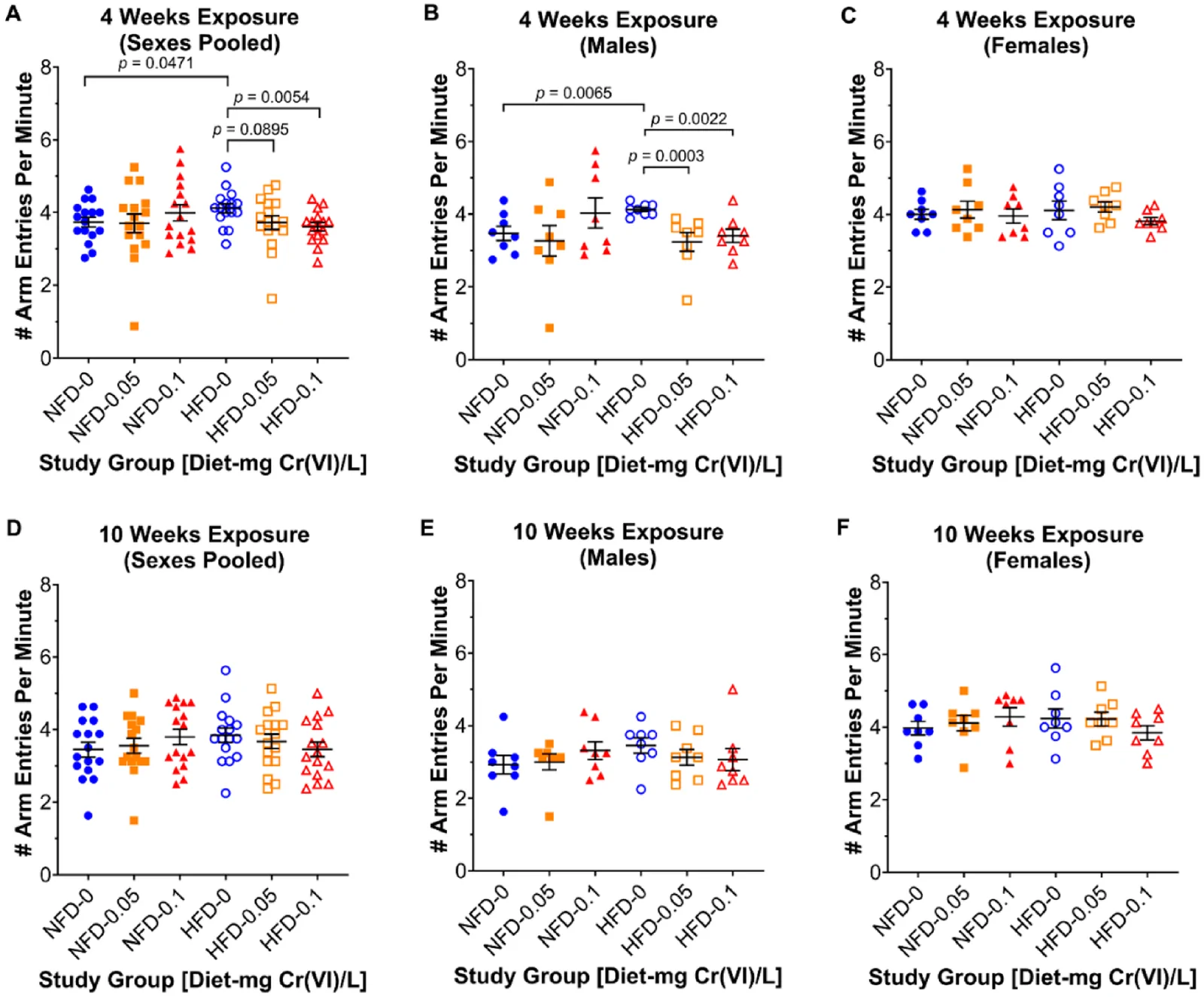
Cr(VI) and high-fat diet co-exposure exhibited a greater impact on activity in rats at earlier time points than later ones. We investigated the effects of co-exposure to Cr(VI) in drinking water and a high-fat diet for 4 and 10 weeks on arm entries per minute in rats. (**A**) Effects of 4-week exposure on arm entries per minute in all rats. (**B**) Effects of 4-week exposure on open arm entries per minute in male rats. (**C**) Effects of 4-week exposure on arm entries per minute in female rats. (**D**) Effects of 10-week exposure on arm entries per minute in all rats. (**E**) Effects of 10-week exposure on arm entries per minute in male rats. (**F**) Effects of 10-week exposure on arm entries per minute in female rats. Data points represent individual rats, and bars represent mean ± SEM. *n* = 8 animals per sex per treatment group for all analyses. Blue circles are control rats, orange squares are 0.05 mg Cr(VI)/L, and red triangles are 0.1 mg Cr(VI)/L; closed data points are normal-fat diet (NFD) and open data points are high-fat diet (HFD). The *p*-values of statistical differences are reported in the graphs.

**Figure 9.**
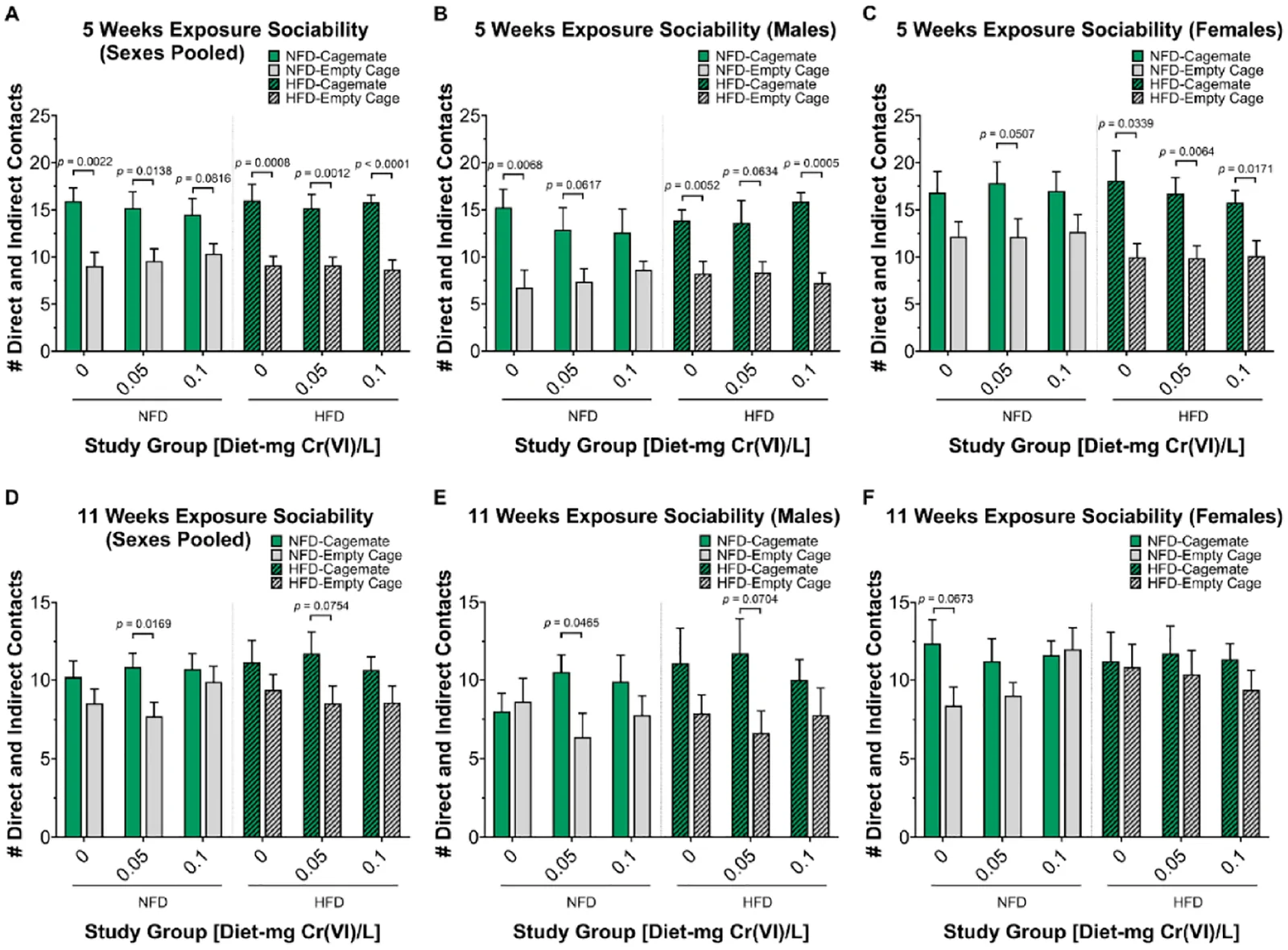
Cr(VI) and high-fat diet co-exposure altered sociability in rats. We investigated the effects of co-exposure to Cr(VI) in drinking water and a high-fat diet for 5 weeks or 11 weeks on the number of interactions with a cagemate vs. an empty cage. (**A**) Effects of 5-week exposure on the number of interactions between the test rat and its cagemate or an empty cage in all rats. (**B**) Effects of 5-week exposure on the number of interactions between the test male rat and its cagemate or an empty cage. (**C**) Effects of 5-week exposure on the number of interactions between the test female rat and its cagemate or an empty cage. (**D**) Effects of 11-week exposure on the number of interactions between the test rat and its cagemate or an empty cage in all rats. (**E**) Effects of 11-week exposure on the number of interactions between the test male rat and its cagemate or an empty cage. (**F**) Effects of 11-week exposure on the number of interactions between the test female rat and its cagemate or an empty cage. Data represent mean ± SEM. For 5-week sociability testing, female sample sizes were *n* = 6, 7, and 6 for the NFD-0, NFD-0.05, and NFD-0.1 groups, respectively; all remaining groups contained *n* = 8 animals. Green bars represent interactions with cagemates, and gray bars represent interactions with empty cage; solid bars are normal-fat diet (NFD), and hashed bars are high-fat diet (HFD). The *p*-values of statistical differences are reported in the graphs.

**Figure 10.**
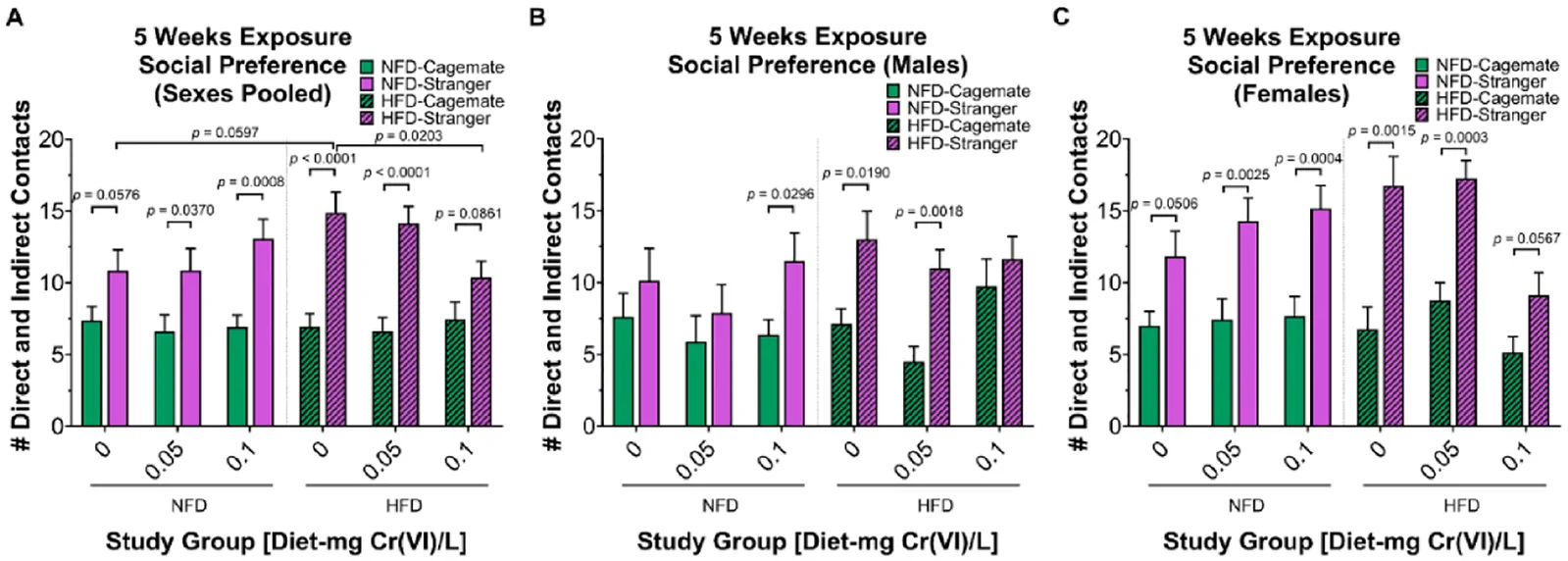

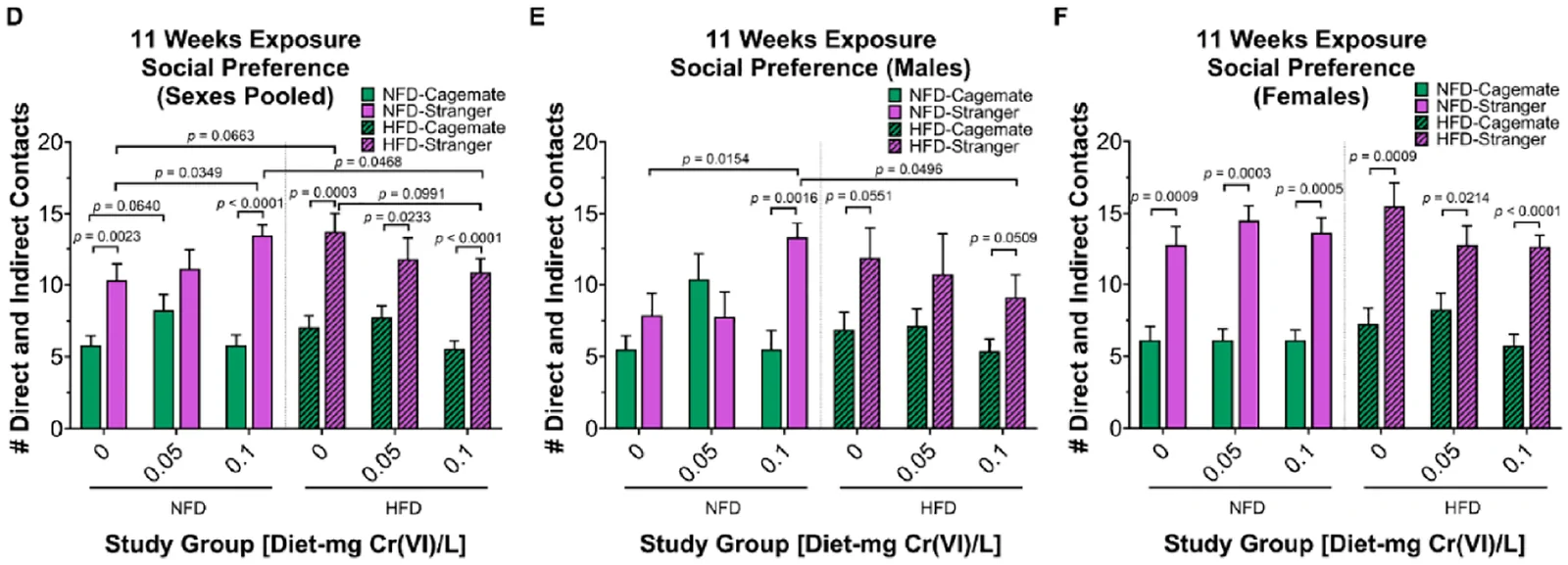
Cr(VI) and high-fat diet co-exposure changed social novelty preference in rats. We investigated the effects of co-exposure to Cr(VI) in drinking water and a high-fat diet for 5 weeks or 11 weeks on social novelty preference in rats. (**A**) Effects of 5-week exposure on the number of interactions between the test rat and its cagemate or a stranger in all rats. (**B**) Effects of 5-week exposure on the number of interactions between the test male rat and its cagemate or a stranger. (**C**) Effects of 5-week exposure on the number of interactions between the test female rat and its cagemate or a stranger. (**D**) Effects of 11-week exposure on the number of interactions between the test rat and its cagemate or a stranger in all rats. (**E**) Effects of 11-week exposure on the number of interactions between the test male rat and its cagemate or a stranger. (**F**) Effects of 11-week exposure on the number of interactions between the test female rat and its cagemate or a stranger. Data represent mean ± SEM. Unless otherwise indicated, *n* = 8 animals per sex per treatment group. For 5-week social novelty preference, female sample sizes were *n* = 6, 7, and 6 for the NFD-0, NFD-0.05, and NFD-0.1 groups, respectively. For 11-week social novelty preference, one outlier (29 contacts) was removed from the NFD-0.1 female group, resulting in *n* = 7 for that group. For male rats, the HFD-0.05 group contained *n* = 7 animals, and one outlier (1 contact) was removed from the NFD-0.1 group after 11 weeks, resulting in *n* = 7 for that group. Green bars represent interactions with cagemates, and purple bars represent interactions with stranger rats; solid bars are normal-fat diet (NFD), and hashed bars are high-fat diet (HFD). The *p*-values of statistical differences are reported in the graphs.

**Figure 11.**
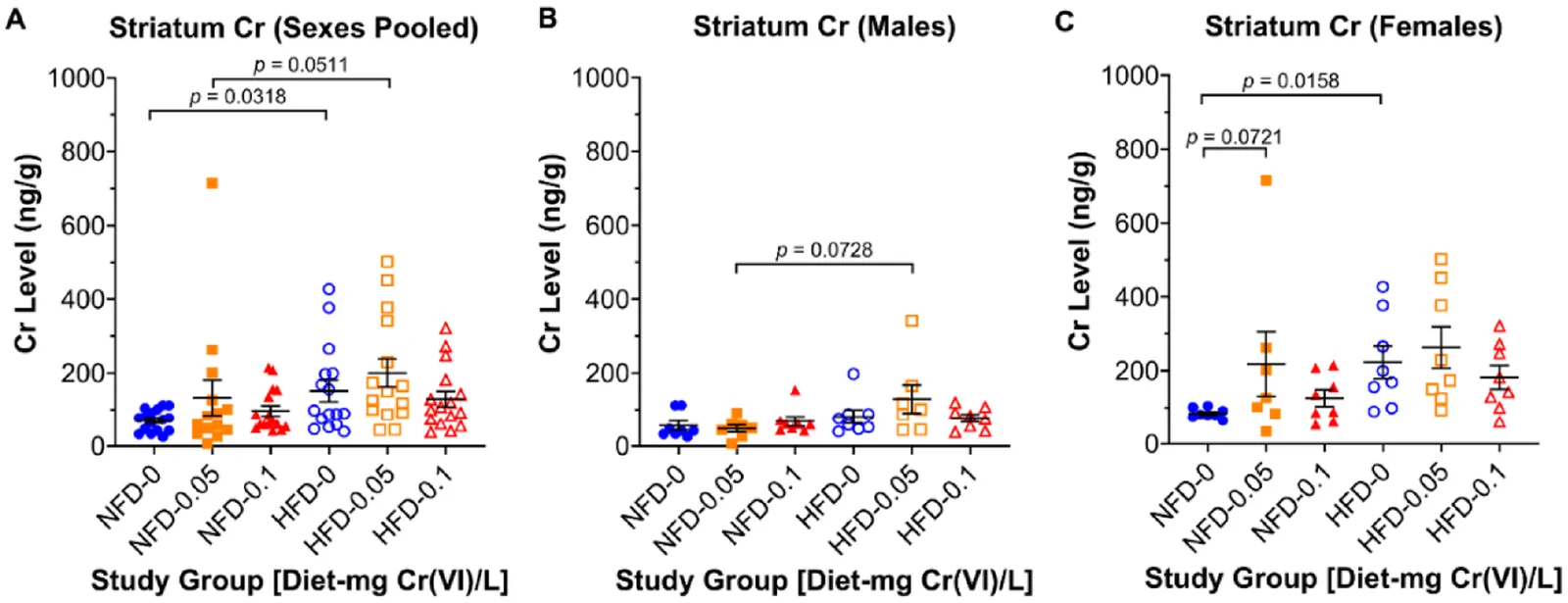

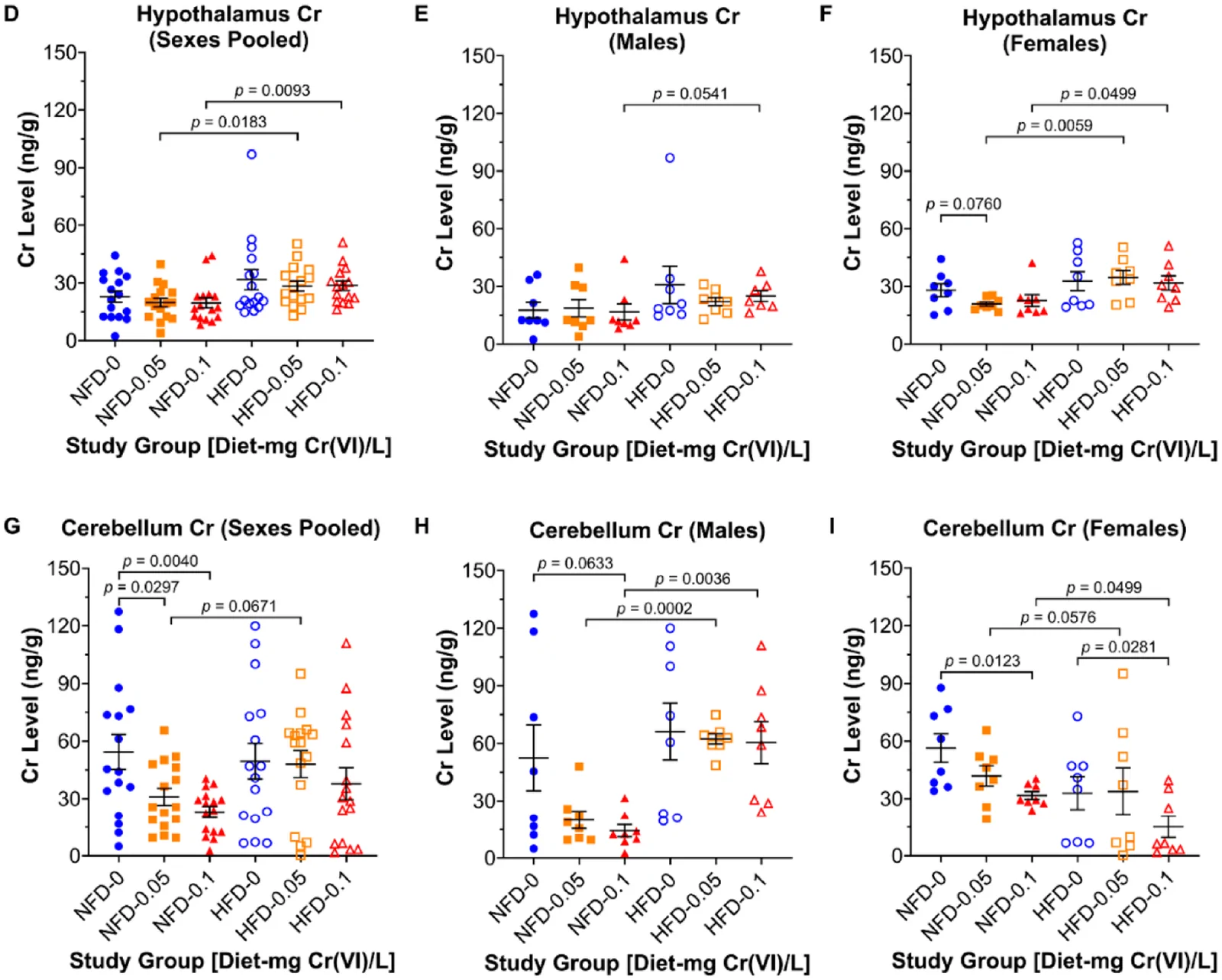
High-fat diet affected Cr accumulation in the striatum, hypothalamus and cerebellum of rats. We measured Cr levels using ICP-MS in striatum, hypothalamus and cerebellum after co-exposure to Cr(VI) in drinking water and a high-fat diet in rats. (**A**) Cr levels in striatum of all rats. (**B**) Cr levels in the striatum of male rats. (**C**) Cr levels in the striatum of female rats. (**D**) Cr levels in the hypothalamus of all rats. (**E**) Cr levels in the hypothalamus of male rats. (**F**) Cr levels in the hypothalamus of female rats. (**G**) Cr levels in the cerebellum of all rats. (**H**) Cr levels in the cerebellum of male rats. (**I**) Cr levels in the cerebellum of female rats. Data points represent individual rats, and bars represent mean ± SEM. Unless otherwise indicated, *n* = 8 animals per sex per treatment group. For the striatum, male sample sizes were *n* = 6 in the NFD + 0.05 mg/L Cr(VI) and HFD + 0.05 mg/L Cr(VI) groups. One striatal value (875.6 ng/g) from a female in the NFD + 0.05 mg/L Cr(VI) group was excluded as an outlier, resulting in *n* = 7 for that group. One hypothalamic value (497.4 ng/g) from a male in the HFD + 0.1 mg/L Cr(VI) group was excluded as an outlier, resulting in *n* = 7 for that group. Removing these outliers did not affect the interpretation of the data. Blue circles are control rats, orange squares are 0.05 mg Cr(VI)/L, and red triangles are 0.1 mg Cr(VI)/L; closed data points are normal-fat diet (NFD), and open data points are high-fat diet (HFD). The *p*-values of statistical differences are reported in the graphs.

**Figure 12.**
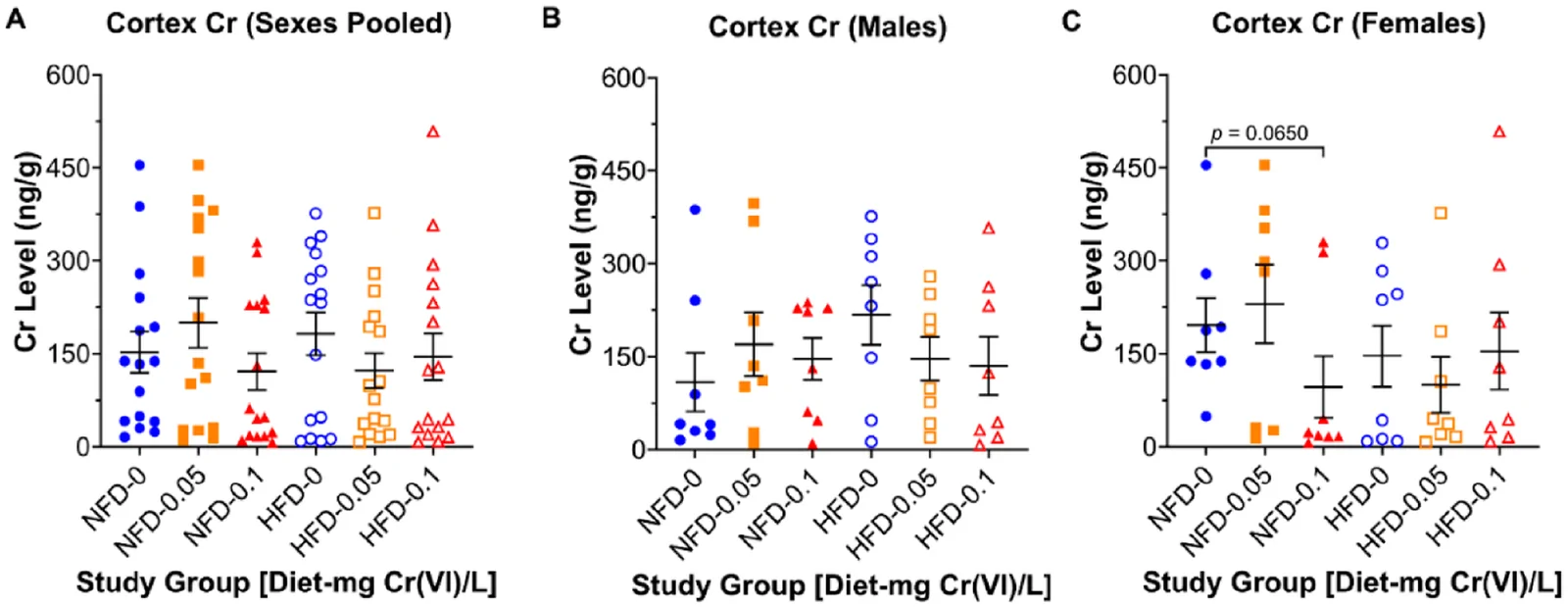

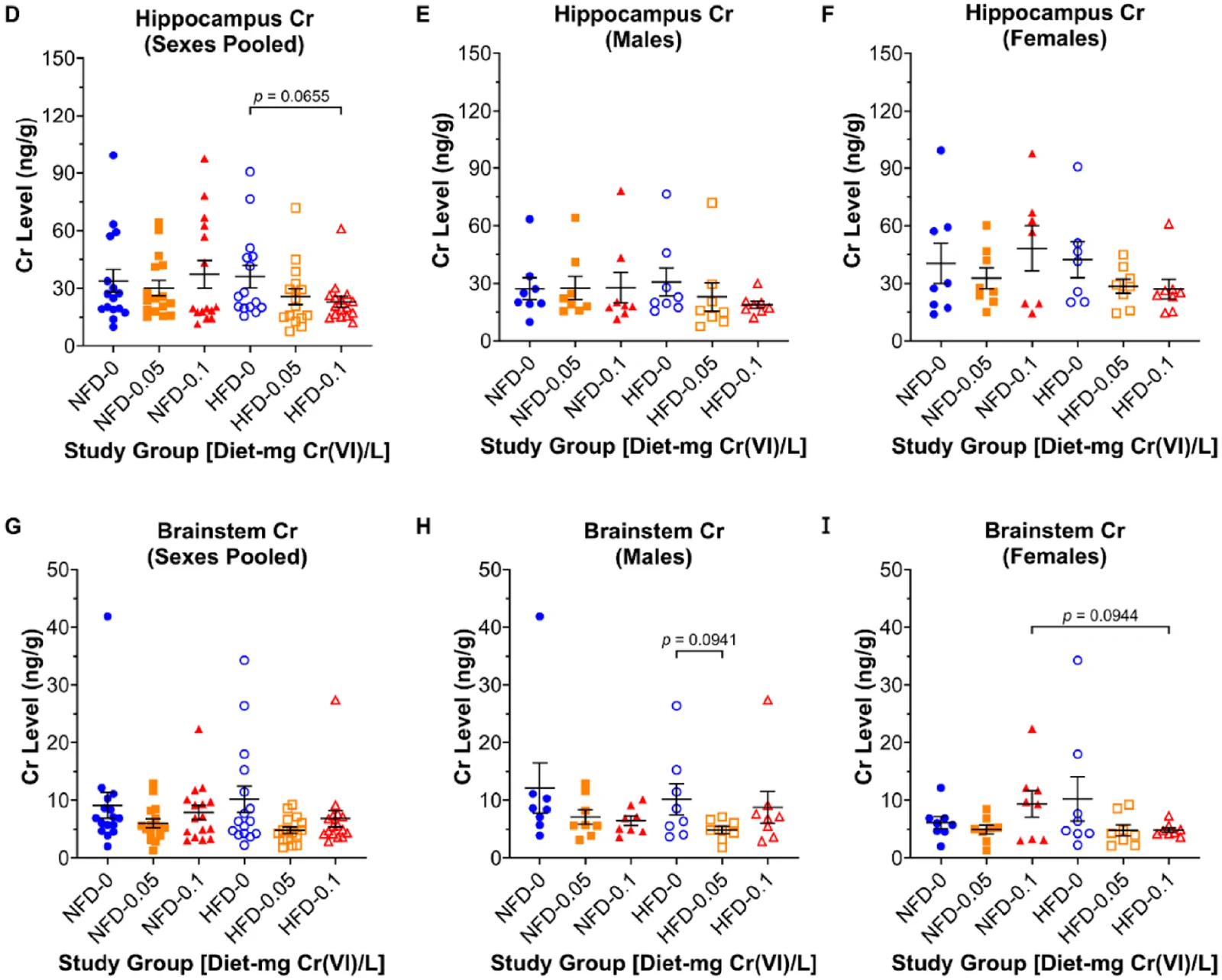
High-fat diet affected Cr accumulation in the cortex, hippocampus and brainstem of rats. We measured Cr levels with ICP-MS in the cortex, hippocampus, and brainstem after co-exposure to Cr(VI) in drinking water and a high-fat diet in rats. (**A**) Cr levels in the cortex of all rats. (**B**) Cr levels in the cortex of male rats. (**C**) Cr levels in the cortex of female rats. (**D**) Cr levels in the hippocampus of all rats. (**E**) Cr levels in the hippocampus of male rats. (**F**) Cr levels in the hippocampus of female rats. (**G**) Cr levels in the brainstem of all rats. (**H**) Cr levels in the brainstem of male rats. (**I**) Cr levels in the brainstem of female rats. Data points represent individual rats, and bars represent mean ± SEM. Unless otherwise indicated, *n* = 8 animals per sex per treatment group. For hippocampal analyses, the female NFD + 0.1 mg/L Cr(VI) group contained *n* = 7 animals. In addition, one hippocampal value (1328.9 ng/g from a female fed high-fat diet alone) was removed as the outlier, resulting in *n* = 7 for that group. Blue circles are control rats, orange squares are 0.05 mg Cr(VI)/L, and red triangles are 0.1 mg Cr(VI)/L; closed data points are normal-fat diet (NFD), and open data points are high-fat diet (HFD). The *p*-values of statistical differences are reported in the graphs.

**Figure 13.**
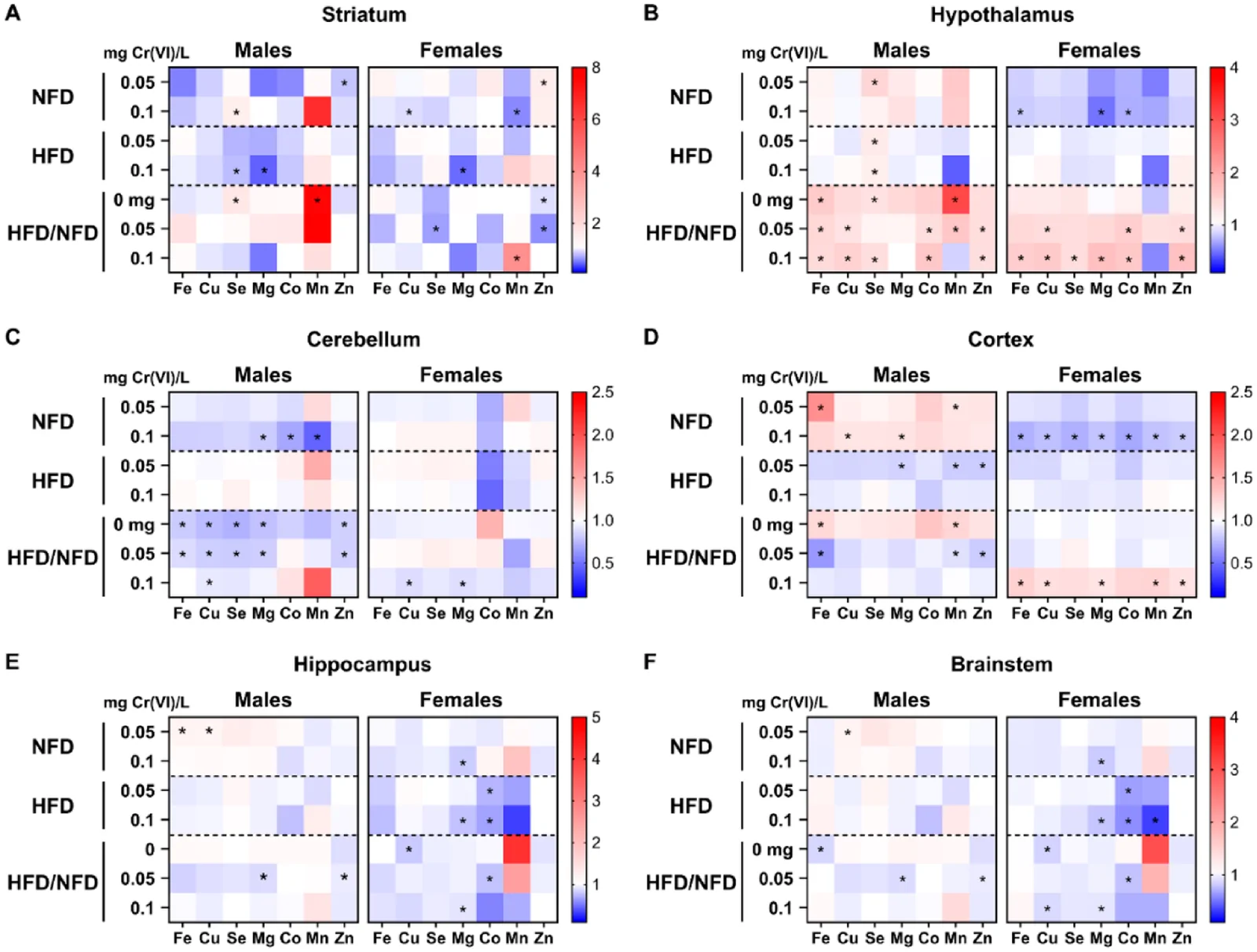
High-fat diet and Cr(VI) disrupted essential metal homeostasis in rat brains. We measured essential metal levels using ICP-MS in striatum, hypothalamus, cerebellum, cortex, hippocampus, and brainstem after co-exposure to Cr(VI) in drinking water and a high-fat diet in rats. (**A**) Essential metal levels in the striatum. (**B**) Essential metal levels in the hypothalamus. (**C**) Essential metal levels in cerebellum. (**D**) Essential metal levels in the cortex. (**E**) Essential metal levels in the hippocampus. (**F**) Essential metal levels in the brainstem. Data are reported as fold-change relative to sex- and diet-matched controls (NFD, HFD), then fold-change in the high-fat diet groups relative to Cr(VI)-matched normal diet groups (HFD/NFD). Red color indicates increased metal levels; blue color indicates decreased metal levels; * *p* < 0.05.

**Table 1.** Timeline of behavioral assays during the 90-day Cr(VI) exposure period.

Behavior Assay	Week #	Behavior(s) Assessed
Grip Strength	1	Neuromuscular function
	7	
Open-Field Assay	2	Exploration, rearing, locomotion
	8	
Elevated Plus Maze	3	Anxiety
	9	
Y-Maze	4	Spatial memory, activity
	10	
3-Chamber Assay	5	Sociability, social novelty preference
	11	

## Data Availability

The original contributions presented in the study are included in the article/Supplementary Material, further inquiries can be directed to the corresponding author.

## Highlights

### What are the main findings?

- Cr(VI) exposure impaired grip strength in rats, and high-fat diet co-exposure attenuated this effect.
- Co-exposure to Cr(VI) with a high-fat diet led to reduced exploratory behavior, motor function, and activity while increasing anxiety.
- Co-exposure to Cr(VI) with a high-fat diet produced modest effects on spatial memory, impacted sociability, and altered social novelty preference.

### What are the implications of the main findings?

- Cr(VI) exposure with high-fat diet co-exposure induced sex-specific behavioral changes.
- High-fat diet altered chromium (Cr) accumulation in the brain in a region- and sex-specific manner.
- Cr(VI) exposure with a high-fat diet disrupted essential metal homeostasis in a sex- and brain region-specific manner.
