# Supplementary material for: Neurobehavioral Responses of Rats Exposed to Low-Concentration Hexavalent Chromium [Cr(VI)] in Drinking Water Combined with a High-Fat Diet
Source: Appl Sci (Basel). Author manuscript; Available in PMC 2026 Oct 17. (PMC13671220; doi:10.3390/app16157367)
Supplement: Supplementary file 1 [file NIHMS2214079-supplement-supplementary_data.pdf]

## Supplementary Information

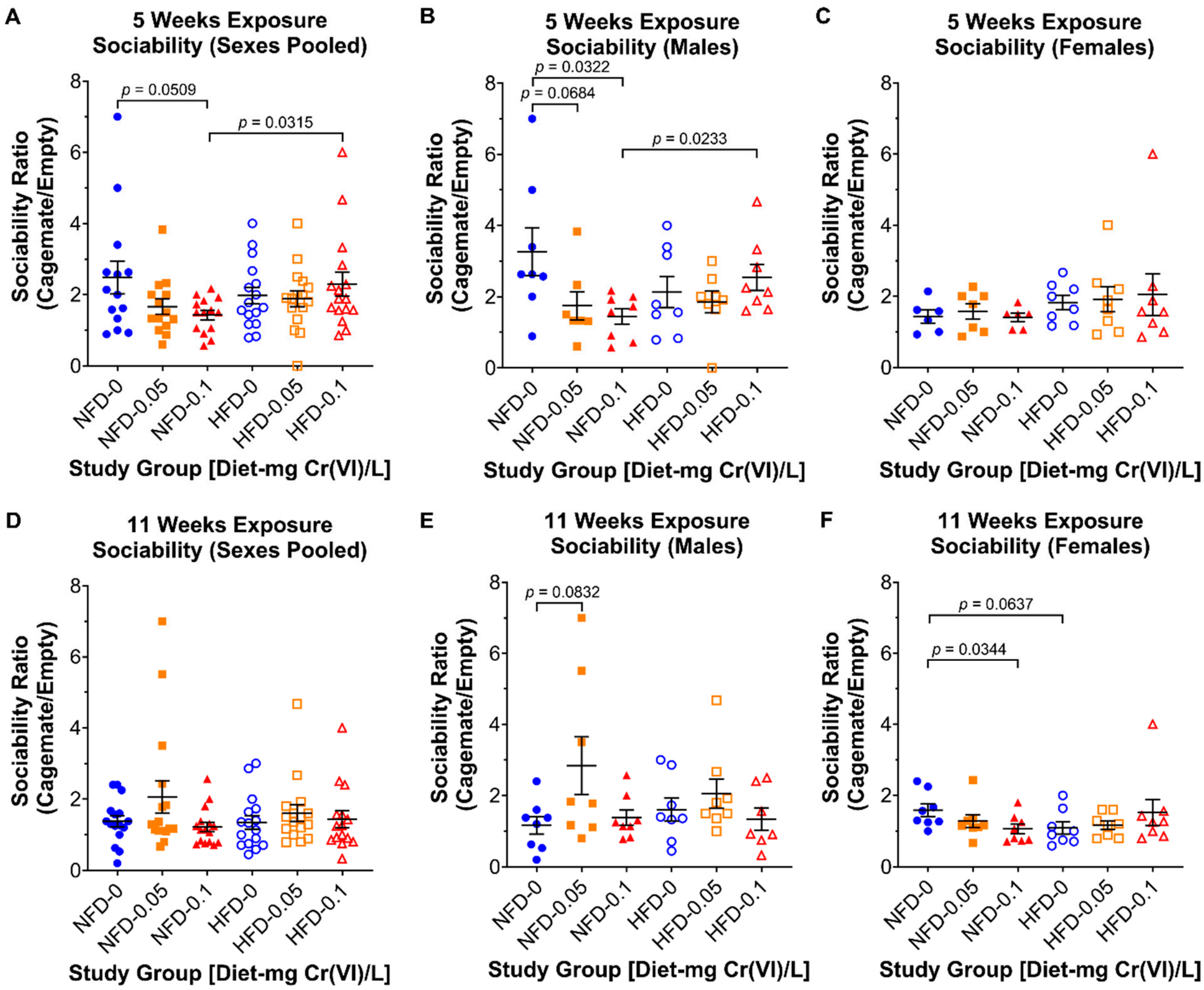


**Suppl. Figure S1. Cr(VI) and high-fat diet co-exposure impacted sociability in rats.** We investigated the effects of co-exposure to Cr(VI) in drinking water and a high-fat diet for 5 and 11 weeks on the ratio of interactions with a cagemate vs. an empty cage. (**A**) Effects of 5-week exposure on the ratio of interactions with a cagemate vs. an empty cage in all rats. (**B**) Effects of 5-week exposure on the ratio of interactions with a cagemate vs. an empty cage in male rats. (**C**) Effects of 5-week exposure on the ratio of interactions with a cagemate vs. an empty cage in female rats. (**D**) Effects of 11-week exposure on the ratio of interactions with a cagemate vs. an empty cage in all rats. (**E**) Effects of 11-week exposure on the ratio of interactions with a cagemate vs. an empty cage in male rats. (**F**) Effects of 11-week exposure on the ratio of interactions with a cagemate vs. an empty cage in female rats. Data points represent individual rats, bars represent mean ± SEM. Blue circles are control rats, orange squares are 0.05 mg Cr(VI)/L, and red triangles are 0.1 mg Cr(VI)/L; closed data points are standard diet (NFD), and open data points are high-fat diet (HFD). The *p*-values of statistical differences are reported in the graphs.

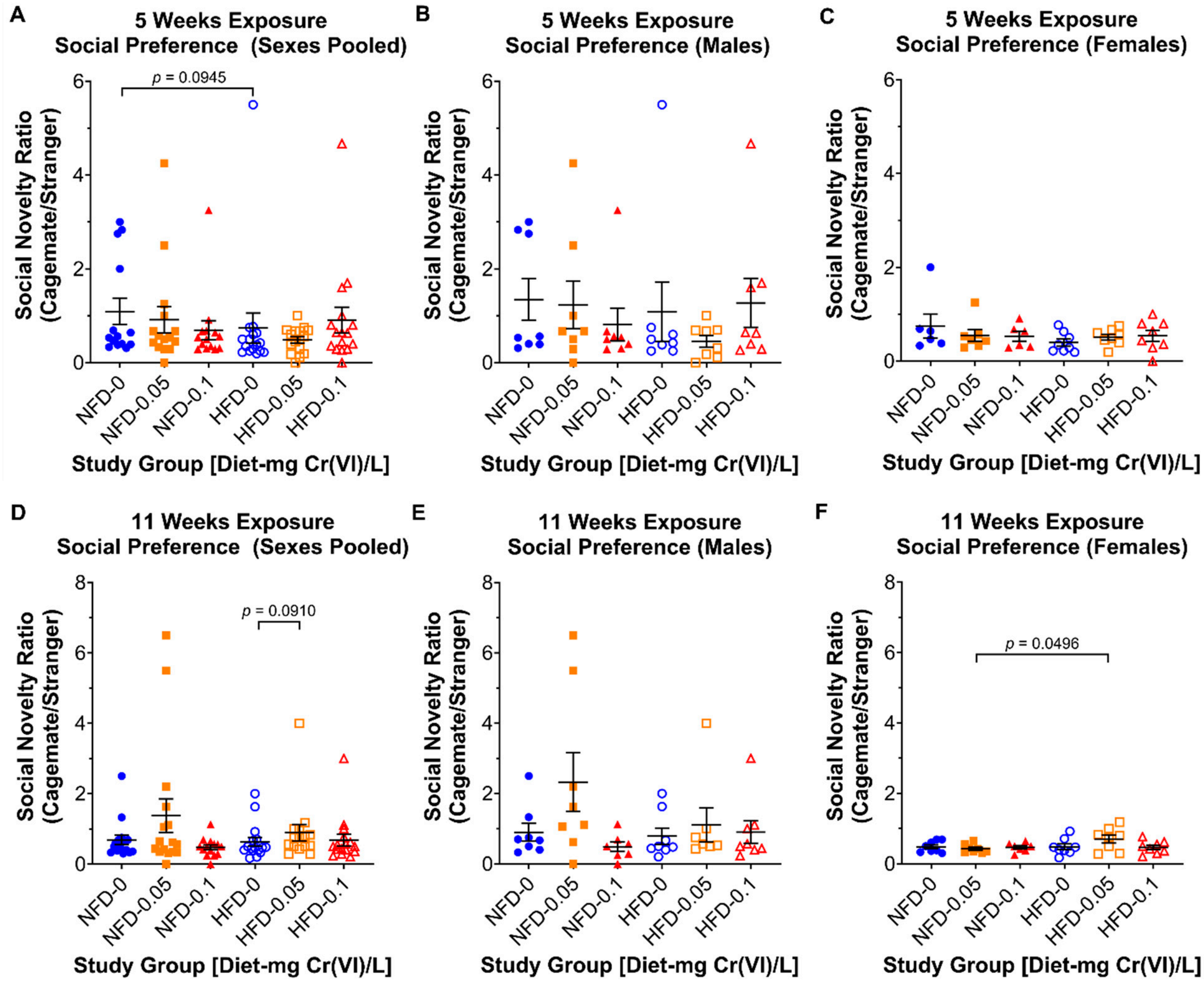


**Suppl. Figure S2. Cr(VI) and high-fat diet co-exposure impacted the social novelty ratio in rats.** We investigated the effects of co-exposure to Cr(VI) in drinking water and a high-fat diet for 5 weeks or 11 weeks on the ratio of interactions with a cagemate vs. a stranger rat. (**A**) Effects of 5-week exposure on the ratio of interactions with a cagemate vs. a stranger in all rats. (**B**) Effects of 5-week exposure on the ratio of interactions with a cagemate vs. a cagemate vs. a stranger in male rats. (**C**) Effects of 5-week exposure on the ratio of interactions with a cagemate vs. a stranger in female rats. (**D**) Effects of 11-week exposure on the ratio of interactions with a cagemate vs. a stranger in all rats. (**E**) Effects of 11-week exposure on the ratio of interactions with a cagemate vs. a stranger in male rats. (**F**) Effects of 11-week exposure on the ratio of interactions with a cagemate vs. a stranger in female rats. Data points represent individual rats, bars represent mean ± SEM. Blue circles are control rats, orange squares are 0.05 mg Cr(VI)/L, and red triangles are 0.1 mg Cr(VI)/L; closed data points are standard diet (NFD), and open data points are high-fat diet (HFD). The *p*-values of statistical differences are reported in the graphs.

## Suppl. Table S1: Two-way ANOVA results for behavioral outcomes.

| Endpoint | Diet F(df) | Diet *p* | Cr F(df) | Cr *p* | Interaction F(df) | Interaction *p* |
|---|---|---|---|---|---|---|
| Grip Strength 1-week | F(1, 90)=1.108 | 0.2954 | F(2, 90)=1.079 | 0.3444 | F(2, 90)=1.963 | 0.1464 |
| Grip Strength 7-week | F(1, 84)=14.15 | 0.0003 | F(2, 84)=1.408 | 0.2504 | F(2, 84)=0.6255 | 0.5375 |
| OFA Distance 2-week | F(1, 86)=0.9243 | 0.3391 | F(2, 86)=0.4832 | 0.6185 | F(2, 86)=4.340 | 0.0160 |
| OFA Distance 8-week | F(1, 90)=7.930 | 0.006 | F(2, 90)=0.3393 | 0.7132 | F(2, 90)=1.523 | 0.2235 |
| OFA Center Exploration 2-week | F(1, 88)=3.275 | 0.0738 | F(2, 88)=0.4195 | 0.6587 | F(2, 88)=1.525 | 0.2233 |
| OFA Center Exploration 8-week | F(1, 89)=0.3562 | 0.5522 | F(2, 89)=2.929 | 0.0586 | F(2, 89)=1.926 | 0.1517 |
| OFA Rearing Count 2-week | F(1, 90)=0.0201 | 0.8876 | F(2, 90)=0.0631 | 0.9389 | F(2, 90)=0.6278 | 0.5361 |
| OFA Rearing Count 8-week | F(1, 90)=0.0074 | 0.9317 | F(2, 90)=0.4418 | 0.6443 | F(2, 90)=0.0344 | 0.9662 |
| EPM Open Arm Exploration 3-week | F(1, 90)=4.063 | 0.0468 | F(2, 90)=0.3374 | 0.7145 | F(2, 90)=2.727 | 0.0708 |
| EPM Open Arm Exploration 9-week | F(1, 90)=0.4032 | 0.527 | F(2, 90)=0.5299 | 0.5905 | F(2, 90)=0.9052 | 0.4081 |
| Y-Maze Non-Alternations 4-week | F(1, 89)=0.0075 | 0.9311 | F(2, 89)=0.4865 | 0.6164 | F(2, 89)=0.0979 | 0.9069 |
| Y-Maze Non-Alternations 10-week | F(1, 89)=0.0162 | 0.899 | F(2, 89)=1.604 | 0.2068 | F(2, 89)=0.7421 | 0.479 |
| Y-Maze Arm Entries 4-week | F(1, 90)=0.0023 | 0.9619 | F(2, 90)=0.7274 | 0.486 | F(2, 90)=2.230 | 0.1135 |
| Y-Maze Arm Entries 10-week | F(1, 90)=0.1196 | 0.7302 | F(2, 90)=0.0118 | 0.9883 | F(2, 90)=1.713 | 0.1862 |
| Sociability Ratio 5-week | F(1, 84)=0.7030 | 0.4042 | F(2, 84)=1.409 | 0.25 | F(2, 84)=2.860 | 0.0629 |
| Social Novelty Ratio 5-week | F(1, 85)=0.8353 | 0.3633 | F(2, 85)=0.3628 | 0.6968 | F(2, 85)=0.9663 | 0.3846 |
| Sociability Ratio 11-week | F(1, 89)=0.1766 | 0.6753 | F(2, 89)=2.466 | 0.0907 | F(2, 89)=0.8682 | 0.4233 |
| Social Novelty Ratio 11-week | F(1, 87)=0.2990 | 0.5859 | F(2, 87)=2.956 | 0.0573 | F(2, 87)=0.9998 | 0.3722 |

OFA: Open-Field Assay, EPM: Elevated Plus Maze

## Suppl. Table S2: Cr(VI) and high-fat diet co-exposure altered essential metal levels in the rat brain.

| Brain Region | Sex | Age | Cr(VI) Concentration [mg/L] | Average Metal Level [ng/g] (SEM) | | | | | | |
|---|---|---|---|---|---|---|---|---|---|---|
| | | | | *Fe* | *Cu* | *Se* | *Mg* | *Co* | *Mn* | *Zn* |
| Striatum | Male | Normal-fat Diet | 0 | 23,311.81 | 3,836.13 | 100.67 | 337,052.32 | 10.74 | 455.64 | 14,731.35 |
| | | | | (4,790.83) | (393.66) | (12.31) | (63,179.12) | (2.06) | (64.57) | (1,122.67) |
| | | | 0.05 | 12,787.01 | 3,164.89 | 109.61 | 176,282.07 | 6.24 | 518.59 | 11628.31[a] |
| | | | | (3,392.02) | (141.61) | (27.69) | (54,334.38) | (1.66) | (161.70) | (304.58) |

| | | | | | | | | | | |
|---|---|---|---|---|---|---|---|---|---|---|
| | | | 0.1 | 18,210.85 | 3,422.16 | 149.50[a] | 339,807.24 | 9.55 | 3,030.55 | 12,833.21 |
| | | | | (1,691.58) | (149.79) | (18.54) | (118,362.12) | (1.42) | (1,014.17) | (680.46) |
| | | **High-fat Diet** | 0 | 21,061.80 | 3,616.48 | 169.13[a] | 403,465.46 | 12.35 | 3449.04[a] | 12,874.68 |
| | | | | (3,945.29) | (313.23) | (10.56) | (142,174.13) | (2.45) | (1,046.12) | (596.25) |
| | | | 0.05 | 23,061.11 | 3,228.55 | 124.05 | 282,815.60 | 10.31 | 4,107.71 | 11,707.71 |
| | | | | (5,783.75) | (185.01) | (18.96) | (59,297.52) | (1.69) | (1,564.49) | (404.49) |
| | | | 0.1 | 19,771.60 | 3,114.82 | 128.81[b] | 177,421.35[b] | 9.90 | 5,778.00 | 12,884.22 |
| | | | | (2,011.45) | (179.59) | (32.20) | (13,099.80) | (1.19) | (1,769.34) | (997.39) |
| | **Female** | **Normal-fat Diet** | 0 | 17,029.29 | 3,941.59 | 145.10 | 325,222.71 | 10.05 | 611.99 | 12,926.01 |
| | | | | (1,311.11) | (128.63) | (22.07) | (72,643.85) | (1.01) | (113.28) | (303.48) |
| | | | 0.05 | 22,506.86 | 3,820.42 | 165.47 | 286,743.21 | 15.58 | 431.19 | 19,744.51[a] |
| | | | | (6,727.06) | (193.80) | (21.67) | (64,045.69) | (4.62) | (35.47) | (2,812.02) |
| | | | 0.1 | 15,534.97 | 3,449.39[a] | 121.33 | 306,935.04 | 10.33 | 353.55[a] | 19,124.47 |
| | | | | (1,420.81) | (111.45) | (15.42) | (100,717.43) | (2.29) | (30.90) | (3,993.28) |
| | | **High-fat Diet** | 0 | 20,871.00 | 3,668.67 | 101.76 | 350,652.40 | 10.11 | 625.23 | 11,405.06[a] |
| | | | | (5,965.42) | (123.37) | (13.34) | (76,287.20) | (0.85) | (122.56) | (490.41) |
| | | | 0.05 | 16,483.20 | 4,198.16 | 108.63[c] | 301,763.94 | 11.10 | 469.34 | 11,840.47[c] |
| | | | | (2,048.11) | (625.99) | (24.99) | (109,889.38) | (1.67) | (60.32) | (682.92) |
| | | | 0.1 | 15,002.31 | 3,147.15 | 126.64 | 168,379.59[b] | 8.46 | 1,444.25[d] | 19,420.83 |
| | | | | (585.13) | (222.25) | (16.57) | (20,926.55) | (1.08) | (729.78) | (6,058.37) |
| **Hypothalamus** | **Male** | **Normal-fat Diet** | 0 | 12,036.12 | 3,543.78 | 122.52 | 194,174.63 | 5.87 | 597.63 | 10,914.97 |
| | | | | (1,570.71) | (482.74) | (16.65) | (50,404.30) | (1.00) | (139.65) | (1,578.37) |
| | | | 0.05 | 13,767.82 | 3,422.98 | 181.79[a] | 245,049.78 | 6.14 | 982.79 | 10,844.08 |
| | | | | (768.84) | (137.46) | (19.61) | (53,158.83) | (0.69) | (437.53) | (321.80) |
| | | | 0.1 | 13,210.35 | 3,441.08 | 143.58 | 264,496.43 | 5.50 | 950.09 | 10,877.76 |
| | | | | (878.74) | (221.07) | (12.40) | (73,250.57) | (0.72) | (414.98) | (339.97) |
| | | **High-fat Diet** | 0 | 19,642.56[a] | 5,035.95 | 166.86[a] | 283,886.60 | 9.25 | 1,867.14[a] | 15,358.62 |
| | | | | (1,778.78) | (585.97) | (8.36) | (37,771.86) | (1.32) | (627.28) | (987.53) |
| | | | 0.05 | 20,186.80[c] | 4619.06[c] | 208.31[b] | 273,739.17 | 8.53[c] | 1,650.26[c] | 15,285.60[c] |
| | | | | (1,160.55) | (213.27) | (10.72) | (30,864.93) | (0.57) | (473.88) | (601.69) |
| | | | 0.1 | 18,988.16[d] | 5,286.36[d] | 199.90[b, d] | 264,704.86 | 9.09[d] | 793.85 | 15,179.51[d] |

| | | | | | | | | | | |
|---|---|---|---|---|---|---|---|---|---|---|
| | | | | (1,085.58) | (416.58) | (19.15) | (18,816.22) | (0.65) | (62.21) | (838.86) |
| | **Female** | **Normal-fat Diet** | 0 | 18,539.66 | 4,447.49 | 158.65 | 324,967.83 | 7.61 | 2,148.49 | 12,416.56 |
| | | | | (1,642.26) | (513.69) | (11.65) | (57,545.03) | (0.84) | (899.25) | (1,127.82) |
| | | | 0.05 | 15,359.94 | 3,940.00 | 135.06 | 203,844.09 | 5.43 | 1,156.48 | 10,916.67 |
| | | | | (338.85) | (273.82) | (13.93) | (24,440.67) | (0.70) | (329.81) | (279.00) |
| | | | 0.1 | 15,009.94[b] | 3,736.71 | 131.17 | 163,841.43[a] | 5.36[a] | 1,465.27 | 10,329.85 |
| | | | | (1,115.48) | (177.32) | (12.76) | (6,647.34) | (0.40) | (921.14) | (230.35) |
| | | **High-fat Diet** | 0 | 23,536.92 | 5,565.06 | 208.54 | 318,536.03 | 8.87 | 1,689.39 | 14,929.23 |
| | | | | (2,557.46) | (556.09) | (20.89) | (38,552.35) | (0.83) | (768.00) | (1,416.47) |
| | | | 0.05 | 22,272.78 | 5,603.99[c] | 189.71 | 297,617.09 | 8.56[c] | 1,578.26 | 15,523.00[c] |
| | | | | (2,136.53) | (553.76) | (22.84) | (46,640.17) | (0.80) | (583.12) | (1,198.79) |
| | | | 0.1 | 23,743.60[d] | 6,093.23[d] | 185.04[d] | 287,186.24[d] | 8.86[d] | 846.16 | 17,775.68[d] |
| | | | | (498.82) | (430.94) | (10.69) | (29,175.93) | (0.46) | (38.30) | (842.62) |
| **Cerebellum** | **Male** | **Normal-fat Diet** | 0 | 41,412.84 | 6,571.16 | 333.52 | 413,685.73 | 13.30 | 2,078.74 | 26,399.95 |
| | | | | (1,462.48) | (419.38) | (24.14) | (27,570.86) | (1.34) | (832.17) | (628.44) |
| | | | 0.05 | 38,723.98 | 5,921.86 | 297.81 | 383,500.30 | 11.53 | 2,536.07 | 25,633.29 |
| | | | | (1,230.09) | (178.41) | (15.21) | (16,357.64) | (0.65) | (1,276.55) | (708.48) |
| | | | 0.1 | 34,234.08 | 5,462.65 | 285.14 | 337,658.21[a] | 9.06[a] | 940.57[a] | 23,581.05 |
| | | | | (3,102.92) | (485.31) | (29.43) | (29,571.16) | (0.99) | (66.79) | (2,101.32) |
| | | **High-fat Diet** | 0 | 33,692.00[a] | 4,989.36[a] | 238.63[a] | 320,430.95[a] | 11.09 | 1,573.82 | 21,933.11[a] |
| | | | | (1,011.27) | (144.62) | (14.11) | (7,737.30) | (0.63) | (295.44) | (855.74) |
| | | | 0.05 | 33,734.71[c] | 4,865.38[c] | 242.47[c] | 316,906.94[c] | 12.13 | 2,354.07 | 21,205.06[c] |
| | | | | (1,239.08) | (167.72) | (16.45) | (9,093.36) | (1.14) | (842.37) | (717.46) |
| | | | 0.1 | 34,549.89 | 4,982.60[d] | 258.91 | 321,562.33 | 10.67 | 1,835.79 | 22,414.21 |
| | | | | (1,584.80) | (125.47) | (20.55) | (12,280.55) | (0.73) | (721.21) | (1,132.40) |
| | **Female** | **Normal-fat Diet** | 0 | 38,190.61 | 5,847.61 | 245.95 | 323,697.50 | 13.88 | 1,906.98 | 21,837.94 |
| | | | | (1,856.57) | (283.91) | (17.00) | (14,259.95) | (3.19) | (535.60) | (967.33) |
| | | | 0.05 | 35,667.64 | 5,582.16 | 230.23 | 309,298.64 | 9.75 | 2,369.33 | 20,575.86 |
| | | | | (2,578.75) | (262.88) | (13.67) | (16,396.36) | (0.87) | (682.67) | (825.96) |
| | | | 0.1 | 38,234.68 | 6,190.85 | 261.31 | 343,252.49 | 10.26 | 1,937.56 | 22,956.56 |
| | | | | (1,270.28) | (191.99) | (15.18) | (10,408.67) | (0.70) | (393.74) | (739.73) |

| | | | | | | | | | | |
|---|---|---|---|---|---|---|---|---|---|---|
| | | **High-fat Diet** | 0 | 35,013.03 | 5,530.09 | 234.20 | 308,847.38 | 20.23 | 1,855.96 | 21,017.81 |
| | | | | (1,638.63) | (220.38) | (14.26) | (13,452.21) | (9.16) | (338.46) | (1,091.99) |
| | | | 0.05 | 36,392.33 | 5,814.38 | 253.28 | 329,340.93 | 10.95 | 1,607.54 | 22,323.26 |
| | | | | (989.29) | (202.93) | (10.63) | (5,815.40) | (0.67) | (380.06) | (472.48) |
| | | | 0.1 | 35,312.51 | 5,414.37[d] | 239.46 | 300,979.76[d] | 9.40 | 1,577.04 | 20,467.91 |
| | | | | (2,114.20) | (271.07) | (9.40) | (15,519.60) | (0.54) | (410.50) | (1,015.87) |
| **Cortex** | **Male** | **Normal-fat Diet** | 0 | 29,261.68 | 5,602.91 | 230.82 | 360,443.01 | 12.10 | 838.55 | 30,835.88 |
| | | | | (1,627.09) | (223.02) | (16.43) | (15,334.12) | (1.46) | (38.78) | (1,578.77) |
| | | | 0.05 | 48,703.18[a] | 6,106.88 | 244.09 | 399,517.90 | 15.61 | 963.66[a] | 35,909.02 |
| | | | | (11,666.81) | (301.54) | (13.47) | (21,187.66) | (1.84) | (42.30) | (1,920.68) |
| | | | 0.1 | 35,859.51 | 6,479.52[a] | 267.79 | 420,944.23[a] | 14.78 | 975.30 | 35,052.91 |
| | | | | (3,262.99) | (274.38) | (16.07) | (18,352.54) | (1.75) | (43.78) | (1,597.95) |
| | | **High-fat Diet** | 0 | 36,134.52[a] | 6,232.24 | 263.70 | 418,039.50 | 16.35 | 1045.32[a] | 35,920.37 |
| | | | | (1,615.88) | (388.42) | (19.92) | (22,736.04) | (1.49) | (53.72) | (1,994.31) |
| | | | 0.05 | 30,850.09[c] | 5,286.25 | 225.77 | 349,978.27[b] | 14.78 | 854.82[b, c] | 29,272.65[b, c] |
| | | | | (2,257.34) | (268.56) | (18.16) | (19,602.62) | (1.49) | (41.42) | (1,658.07) |
| | | | 0.1 | 32,971.79 | 5,801.67 | 271.65 | 398,533.52 | 13.24 | 963.40 | 32,823.17 |
| | | | | (2,703.20) | (338.38) | (20.86) | (30,239.37) | (1.17) | (79.57) | (2,696.93) |
| | **Female** | **Normal-fat Diet** | 0 | 38,903.53 | 7,070.24 | 268.99 | 401,936.91 | 15.94 | 993.33 | 34,587.69 |
| | | | | (2,891.59) | (490.98) | (18.12) | (25,425.31) | (1.52) | (67.86) | (2,285.16) |
| | | | 0.05 | 35,027.71 | 6,286.39 | 220.68 | 362,614.83 | 13.29 | 892.43 | 31,507.76 |
| | | | | (3,105.61) | (411.08) | (22.70) | (21,118.32) | (1.67) | (47.85) | (1,444.29) |
| | | | 0.1 | 27,802.61[a] | 5,452.01[a] | 191.90[a] | 316,329.41[a] | 10.91[a] | 775.07[a] | 28,198.87[a] |
| | | | | (1,572.87) | (429.05) | (18.87) | (22,351.74) | (1.68) | (60.66) | (2,029.73) |
| | | **High-fat Diet** | 0 | 36,240.35 | 7,052.58 | 256.30 | 398,561.34 | 15.01 | 943.78 | 33,030.67 |
| | | | | (1,851.78) | (338.55) | (13.07) | (14,322.72) | (1.35) | (39.68) | (1,191.52) |
| | | | 0.05 | 31,208.32 | 6,032.03 | 239.17 | 362,314.61 | 12.15 | 875.22 | 30,413.32 |
| | | | | (1,712.39) | (363.45) | (16.59) | (18,634.65) | (1.02) | (49.71) | (1,322.66) |
| | | | 0.1 | 35,453.91[d] | 6,490.49[d] | 231.01 | 367,971.37[d] | 13.56 | 978.11[d] | 33,141.55[d] |
| | | | | (1,297.20) | (174.61) | (7.65) | (6,175.44) | (1.17) | (40.31) | (600.70) |
| **Hippocampus** | **Male** | | 0 | 21,715.13 | 4,759.98 | 180.08 | 292,145.64 | 9.99 | 679.28 | 32,171.57 |

| | | | | | | | | | | |
|---|---|---|---|---|---|---|---|---|---|---|
| | | | | (1,513.63) | (284.55) | (22.51) | (25,170.10) | (1.30) | (96.14) | (4,942.60) |
| | | Normal-fat Diet | 0.05 | 26,055.12[a] | 5,466.17[a] | 233.07 | 352,197.50 | 10.63 | 622.94 | 31,163.33 |
| | | | | (1,495.33) | (177.37) | (10.03) | (28,417.07) | (1.14) | (88.57) | (630.80) |
| | | | 0.1 | 22,952.19 | 5,275.70 | 190.16 | 323,197.21 | 8.74 | 654.40 | 30,032.04 |
| | | | | (628.54) | (211.55) | (7.77) | (18,114.48) | (0.47) | (35.45) | (1,201.43) |
| | | High-fat Diet | 0 | 23,684.82 | 5,122.24 | 185.26 | 323,706.03 | 11.04 | 740.62 | 28,276.76 |
| | | | | (875.53) | (245.18) | (11.04) | (14,279.92) | (1.13) | (60.01) | (994.07) |
| | | | 0.05 | 21,797.26 | 4,813.31 | 209.99 | 306,507.16[c] | 10.70 | 644.02 | 28,756.73[c] |
| | | | | (1,597.53) | (385.23) | (17.58) | (30,558.18) | (1.53) | (47.45) | (3,256.96) |
| | | | 0.1 | 22,577.21 | 4,919.65 | 192.22 | 307,656.23 | 8.57 | 937.45 | 27,506.53 |
| | | | | (598.20) | (209.07) | (10.29) | (14,400.97) | (0.65) | (268.95) | (1,002.62) |
| | Female | Normal-fat Diet | 0 | 29,232.05 | 6,363.88 | 196.81 | 366,401.36 | 11.86 | 468.98 | 29,337.69 |
| | | | | (1,049.83) | (349.98) | (7.01) | (22,277.44) | (1.87) | (135.09) | (1,029.51) |
| | | | 0.05 | 28,590.22 | 5,788.83 | 199.71 | 348,172.84 | 10.84 | 517.94 | 28,867.83 |
| | | | | (2,781.64) | (183.50) | (11.78) | (18,855.42) | (0.84) | (113.12) | (924.94) |
| | | | 0.1 | 25,638.47 | 5,827.51 | 184.10 | 298,794.57[a] | 13.86 | 915.36 | 26,428.17 |
| | | | | (1,164.65) | (279.16) | (14.07) | (12,406.27) | (3.97) | (135.30) | (1,098.80) |
| | | High-fat Diet | 0 | 29,920.35 | 5,145.97[a] | 183.22 | 346,283.61 | 11.56 | 1,960.34 | 26,260.99 |
| | | | | (5,090.46) | (198.38) | (14.71) | (21,895.40) | (1.56) | (803.82) | (870.00) |
| | | | 0.05 | 24,594.14 | 5,460.66 | 182.73 | 329,815.82 | 8.49[b, c] | 1,308.74 | 27,008.96 |
| | | | | (685.59) | (197.63) | (13.25) | (23,757.75) | (0.26) | (326.87) | (620.65) |
| | | | 0.1 | 22,866.52 | 5,007.63 | 169.86 | 269,398.53[b, d] | 7.74[b] | 640.83 | 26,548.96 |
| | | | | (466.45) | (85.18) | (9.43) | (9,697.48) | (0.37) | (39.72) | (418.06) |
| **Brainstem** | **Male** | **Normal-fat Diet** | 0 | 13,556.56 | 4,759.98 | 180.08 | 292,145.64 | 9.99 | 679.28 | 32,171.57 |
| | | | | (380.35) | (284.55) | (22.51) | (25,170.10) | (1.30) | (96.14) | (4,942.60) |
| | | | 0.05 | 12,777.24 | 5,466.17[a] | 233.07 | 352,197.50 | 10.63 | 679.74 | 31,163.33 |
| | | | | (609.72) | (177.37) | (10.03) | (28,417.07) | (1.14) | (34.40) | (630.80) |
| | | | 0.1 | 12,600.37 | 5,275.70 | 190.16 | 323,197.21 | 8.74 | 654.40 | 30,032.04 |
| | | | | (405.98) | (211.55) | (7.77) | (18,114.48) | (0.47) | (35.45) | (1,201.43) |
| | | **High-fat Diet** | 0 | 11,560.06[a] | 5,122.24 | 185.26 | 323,706.03 | 11.04 | 740.62 | 28,276.76 |
| | | | | (806.29) | (245.18) | (11.04) | (14,279.92) | (1.13) | (60.01) | (994.07) |

| | | | | | | | | | |
|---|---|---|---|---|---|---|---|---|---|
| | | 0.05 | 12,872.95 | 4,813.31 | 209.99 | 306,507.16[c] | 10.70 | 644.02 | 28,756.73[c] |
| | | | (426.61) | (385.23) | (17.58) | (30,558.18) | (1.53) | (47.45) | (3,256.96) |
| | | 0.1 | 13,385.42 | 4,919.65 | 192.22 | 307,656.23 | 8.57 | 937.45 | 27,506.53 |
| | | | (274.27) | (209.07) | (10.29) | (14,400.97) | (0.65) | (268.95) | (1,002.62) |
| **Female** | **Normal-fat Diet** | 0 | 14,165.27 | 6,363.88 | 196.81 | 366,401.36 | 11.86 | 630.68 | 29,337.69 |
| | | | (613.03) | (349.98) | (7.01) | (22,277.44) | (1.87) | (59.50) | (1,029.51) |
| | | 0.05 | 13,064.03 | 5,788.83 | 199.71 | 348,172.84 | 10.84 | 688.92 | 28,867.83 |
| | | | (736.60) | (183.50) | (11.78) | (18,855.42) | (0.84) | (56.15) | (924.94) |
| | | 0.1 | 12,924.55 | 5,796.10 | 186.58 | 297,390.77[a] | 10.93 | 904.77 | 26,531.22 |
| | | | (631.77) | (243.80) | (12.43) | (10,835.47) | (2.34) | (117.65) | (957.15) |
| | **High-fat Diet** | 0 | 14,009.83 | 5,338.63[a] | 191.34 | 340,732.79 | 12.96 | 1,933.95 | 26,550.28 |
| | | | (369.39) | (258.14) | (15.11) | (19,757.73) | (1.94) | (656.85) | (807.07) |
| | | 0.05 | 13,409.01 | 5,460.66 | 182.73 | 329,815.82 | 8.49[b, c] | 1,308.74 | 27,008.96 |
| | | | (376.26) | (197.63) | (13.25) | (23,757.75) | (0.26) | (326.87) | (620.65) |
| | | 0.1 | 14,144.52 | 5,007.63[d] | 169.86 | 269,398.53[b, d] | 7.74[b] | 640.83[b] | 26,548.96 |
| | | | (489.92) | (85.18) | (9.43) | (9,697.48) | (0.37) | (39.72) | (418.06) |

Data reported as mean (SEM) (ng/g). [a]$p < 0.05$ compared to normal-fat diet control, [b]$p < 0.05$ compared to high-fat diet control, [c]$p < 0.05$ compared to normal-fat diet 0.05 mg/L Cr(VI), [d]$p < 0.05$ compared to normal-fat diet 0.1 mg/L Cr(VI).
